# Supplementary material for: Genome-wide association study results for educational attainment aid in identifying genetic heterogeneity of schizophrenia
Source: Nat Commun. 2018 Aug 6;9:3078. doi: 10.1038/s41467-018-05510-z (PMC6079028; doi:10.1038/s41467-018-05510-z)
Supplement: Supplementary file 1 — Supplementary Information [file 41467_2018_5510_MOESM1_ESM.docx]

##### **Supplementary Figures and Notes for Bansal *et al:***

##### ***“*Genome-wide association study results for educational attainment aid in identifying genetic heterogeneity of schizophrenia*”***

# Supplementary Figures

Supplementary Figure 1: GWAS *Z*-scores for educational attainment and schizophrenia from non-overlapping sample.


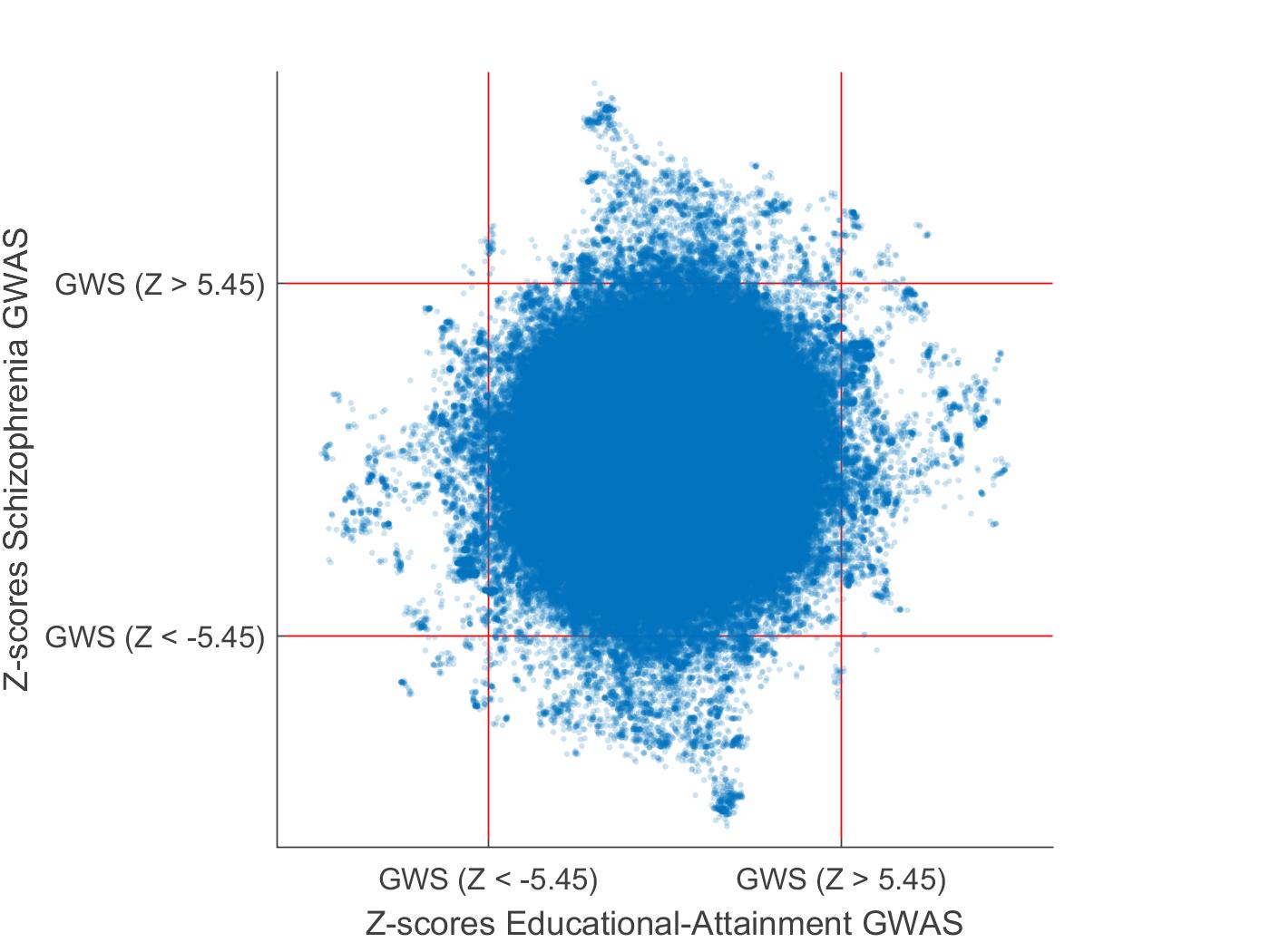


*Notes:* GWAS *Z*-scores for educational attainment (horizontal axis) and schizophrenia (vertical axis) from non-overlapping samples, without pruning for linkage disequilibrium. The red lines indicate the thresholds for univariate genome-wide significance.

Supplementary Figure 2: Scatter plots of *Z*-scores from a GWAS on two simulated (related) traits.

*Notes:* Scatter plots of *Z*-scores from a GWAS on two simulated (related) traits, *A* and *B*. Columns: different proportions of causal loci shared between traits (*π_AB_*). Rows: different correlations of the effects of shared loci (*ρ*). Last row: 40% of shared loci have *ρ* = 1, 40% have *ρ* = −1, and 20% have *ρ* = 0. For each simulation: 25k independent SNPs of which 10% (2.5k) causal, *h*^2^ = 50%, *N*(GWAS) = 25k. Δ*R*^2^ shows the average incremental *R*^2^ over 100 additional simulations of a model predicting *A* in a sample of *N*(hold-out) = 5k individuals, using the PGSs for *A* and *B* as baseline, when splitting up the PGS for *A* by sign-concordance of SNP-effect estimates of *A* and *B*.

Supplementary Figure 3: **LocusZoom plots for high, medium and low pleiotropic SNPs.**

**a. Concordant SNP with high probability of pleiotropy**

**
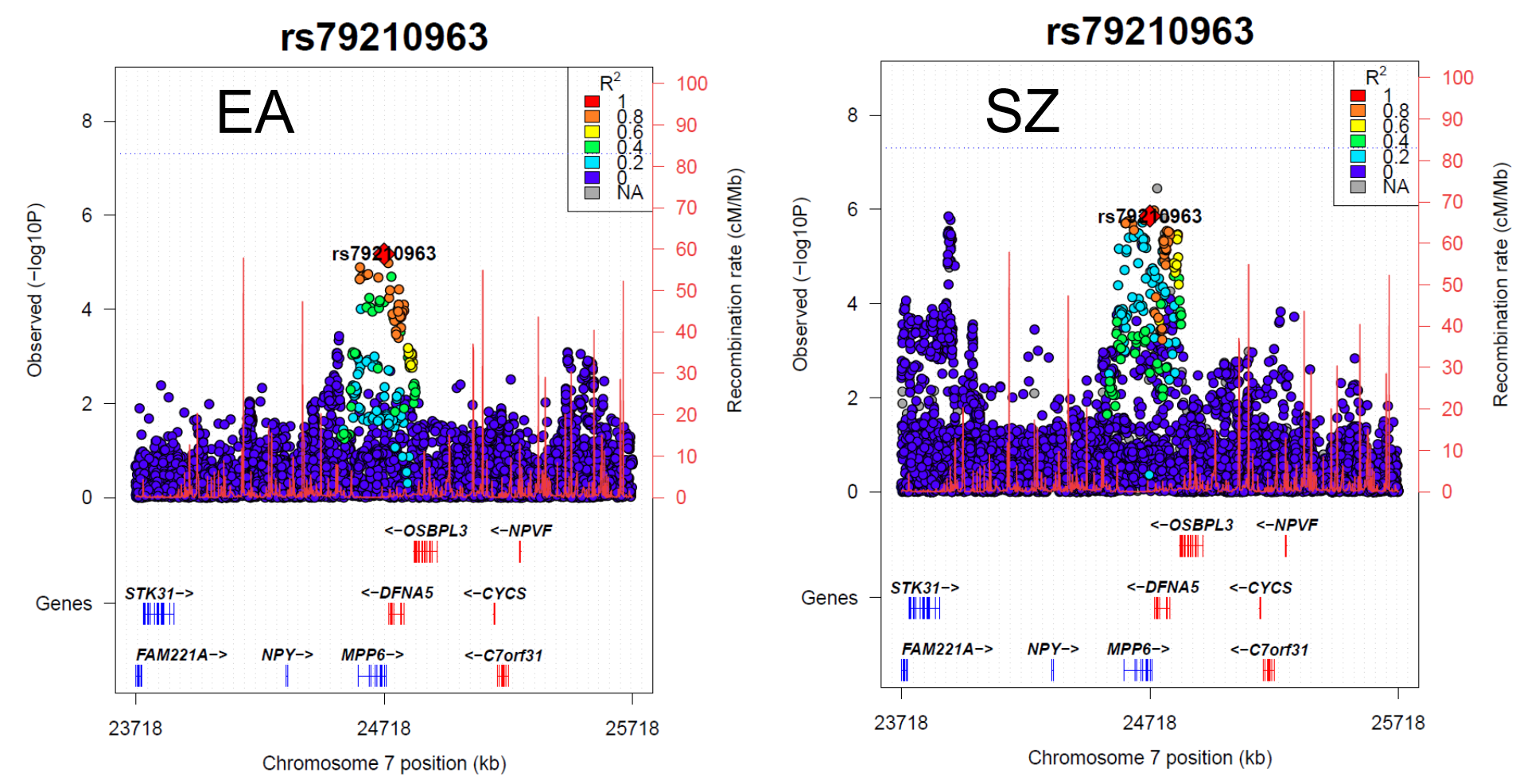
**

**b. Discordant SNP with medium probability of pleiotropy**

**
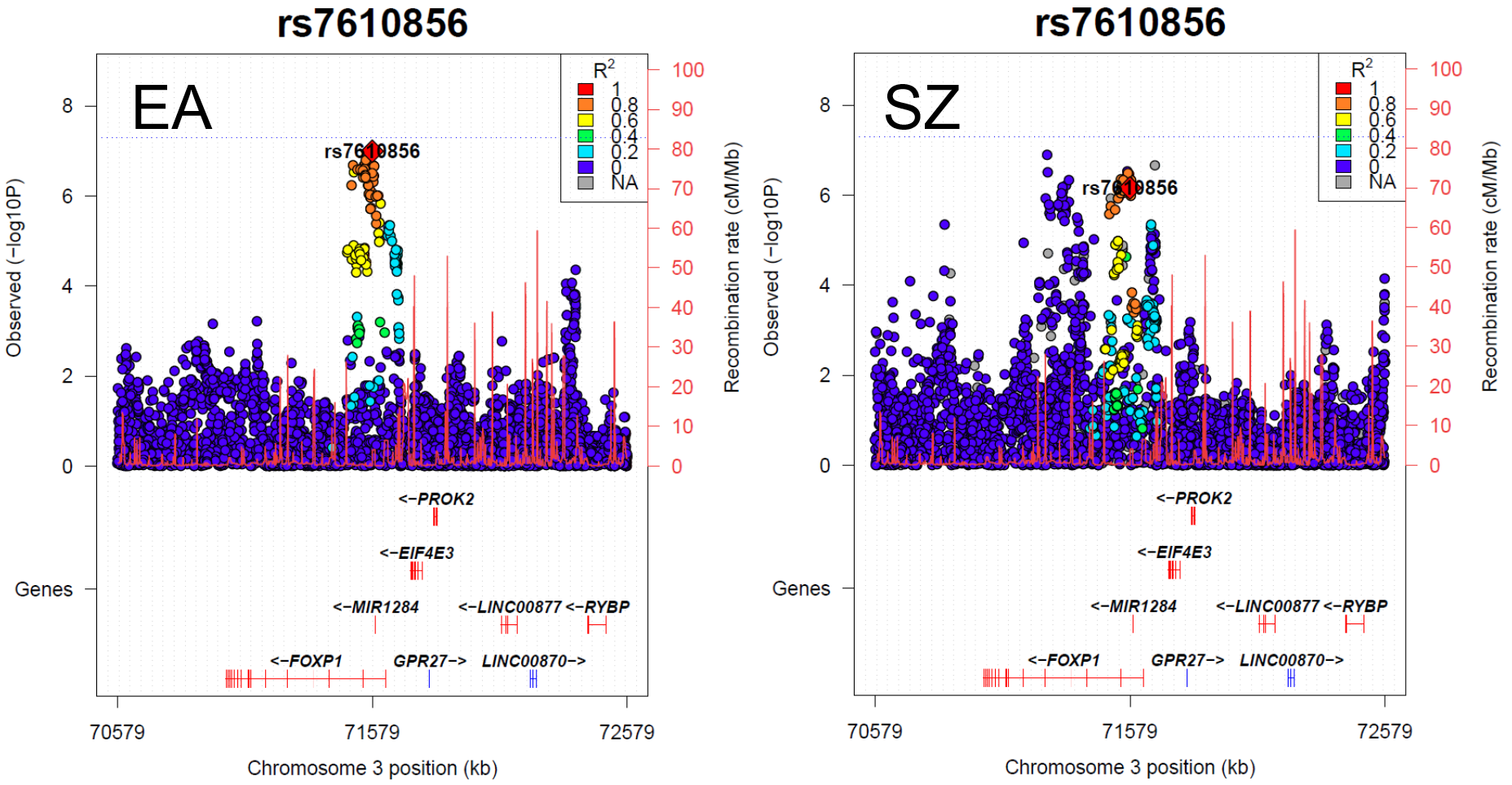
**

**c. SNP with low probability of pleiotropy**

**
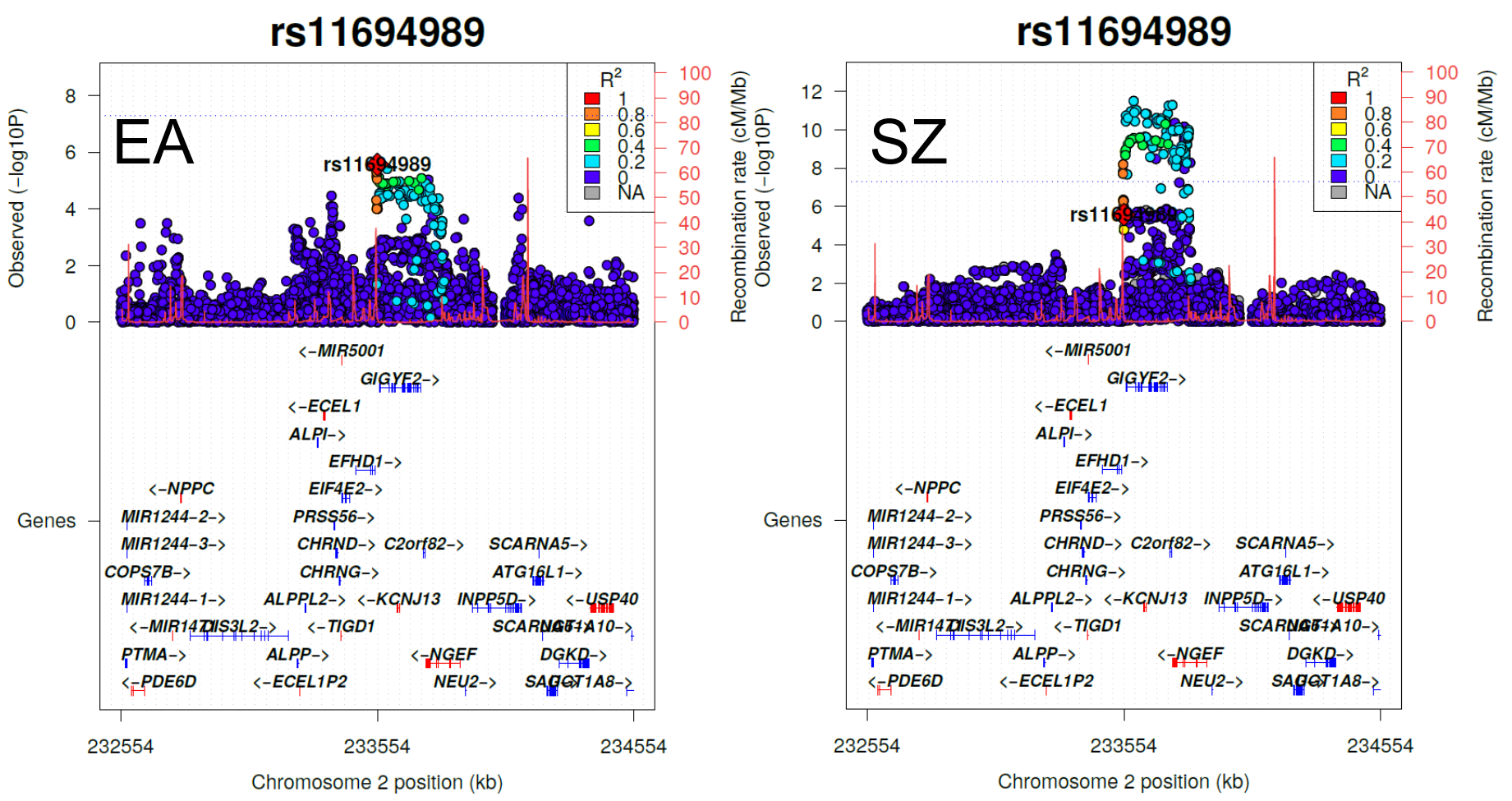
**

*Notes*: Panel a: An example of a concordant SNP that is likely to have pleiotropic effects on SZ and EA. Panel b: Discordant SNP that is likely to have pleiotropic effects on SZ and EA. Panel c: SNP with low probability of pleiotropy. Pleiotropic effects were defined using PAINTOR.

Supplementary Figure 4: Biological annotation of SNPs that are jointly associated with EA ${(P}_{EA}<{10}^{-5})$ and SZ $(P_{SZ}<0.05$).

1. **Gene set enrichment using DEPICT**

| 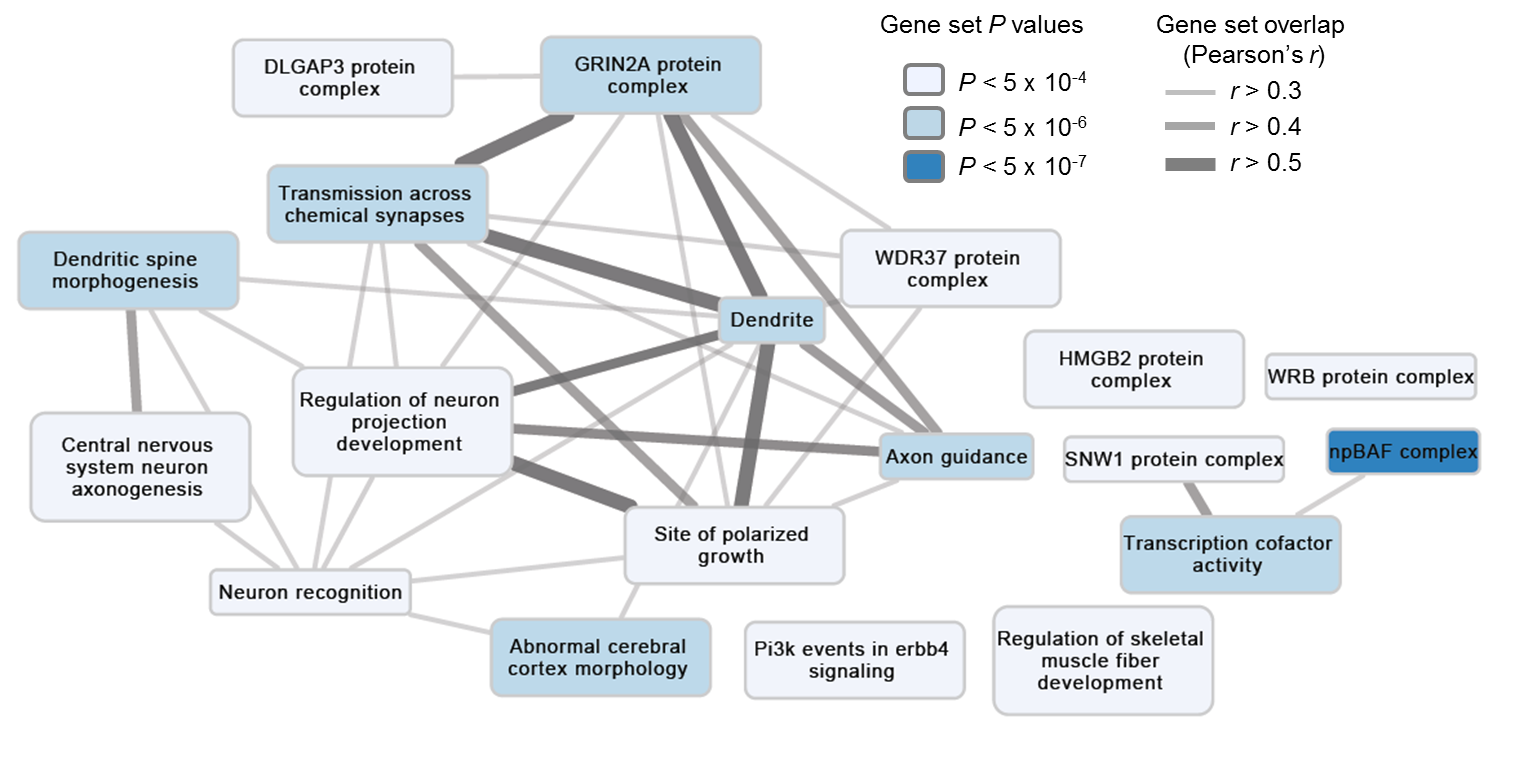 |
| --- |
| 1. **Tissue enrichment using DEPICT** |
| 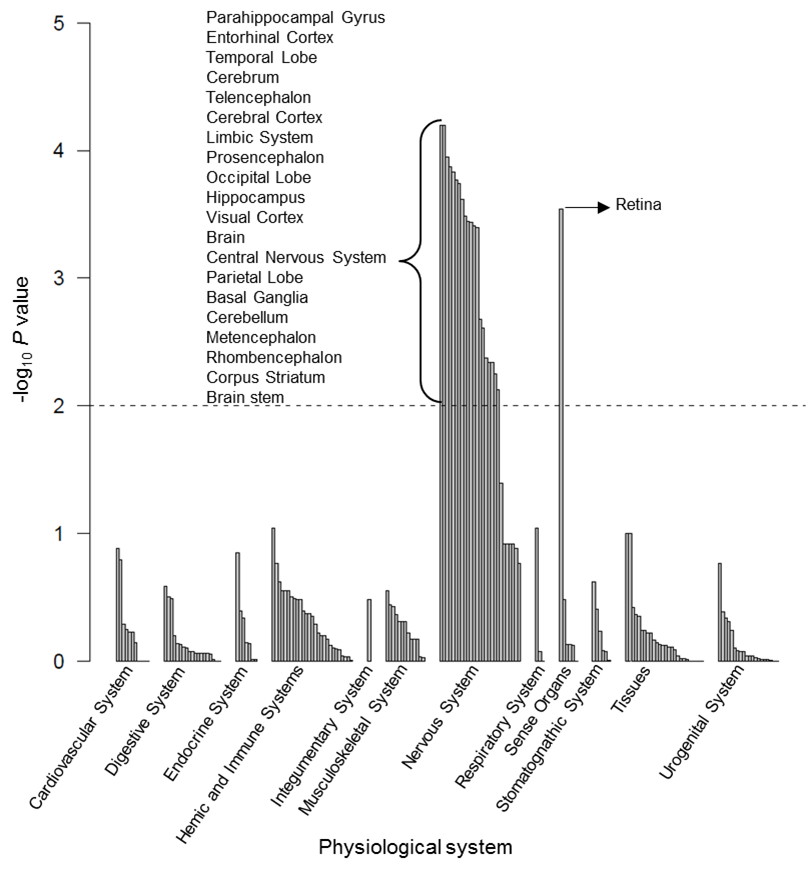 |
|  |

*Notes*: Panel a: Exemplar gene sets identified using DEPICT. Color represents the DEPICT gene set enrichment *P* value (lower *P* values are reflected by darker colors) Gene sets with Pearson correlations above *r* = 0.3 are connected by edges. Panel b: Tissue enrichment results obtained by DEPICT. Names of the 21 significantly enriched tissues are shown above the dashed line.

Supplementary Figure 5: LD-aware enrichment across traits for SNPs that are jointly associated with EA ${(P}_{EA}<{10}^{-5})$ and SZ $(P_{SZ}<0.05$).

**
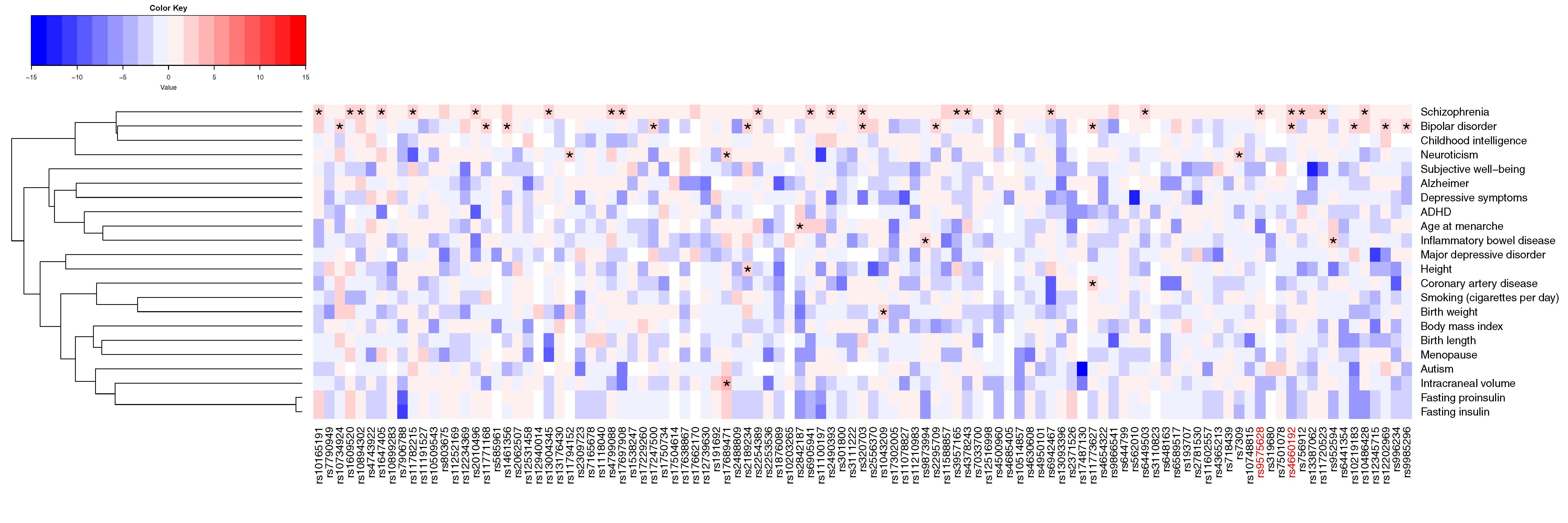
**

*Notes:* Color codes illustrate the degree of LD-aware enrichment. Red and blue represent stronger and weaker LD-aware enrichment than expected, respectively. Darker colors illustrate more substantial deviation from expectation. A star (*) indicates that the observed LD-aware enrichment is significant after FDR correction. Note that the *p*-values of this test are not the same as those reported in the GWAS and proxy-phenotype analyses because the hypothesis that is tested here is conditional on the LD-score of each tested SNP. The lines on the left side represent hierarchical clustering using the euclidean distance and the complete agglomeration method. Note that we restricted our analysis to HapMap3 SNPs because these are available in most publicly available GWAS summary statistics. If a candidate SNP from our proxy-phenotype results was not included in HapMap3, we replaced it by the best available proxy SNP that was available (${LD}_{r^{2}}>0.8$ and a maximum distance of 500 kb to our missing EA lead-SNPs, choosing the proxy with the highest ${LD}_{r^{2}}$).

Supplementary Figure 6: Allele frequency distribution of clumped SNPs


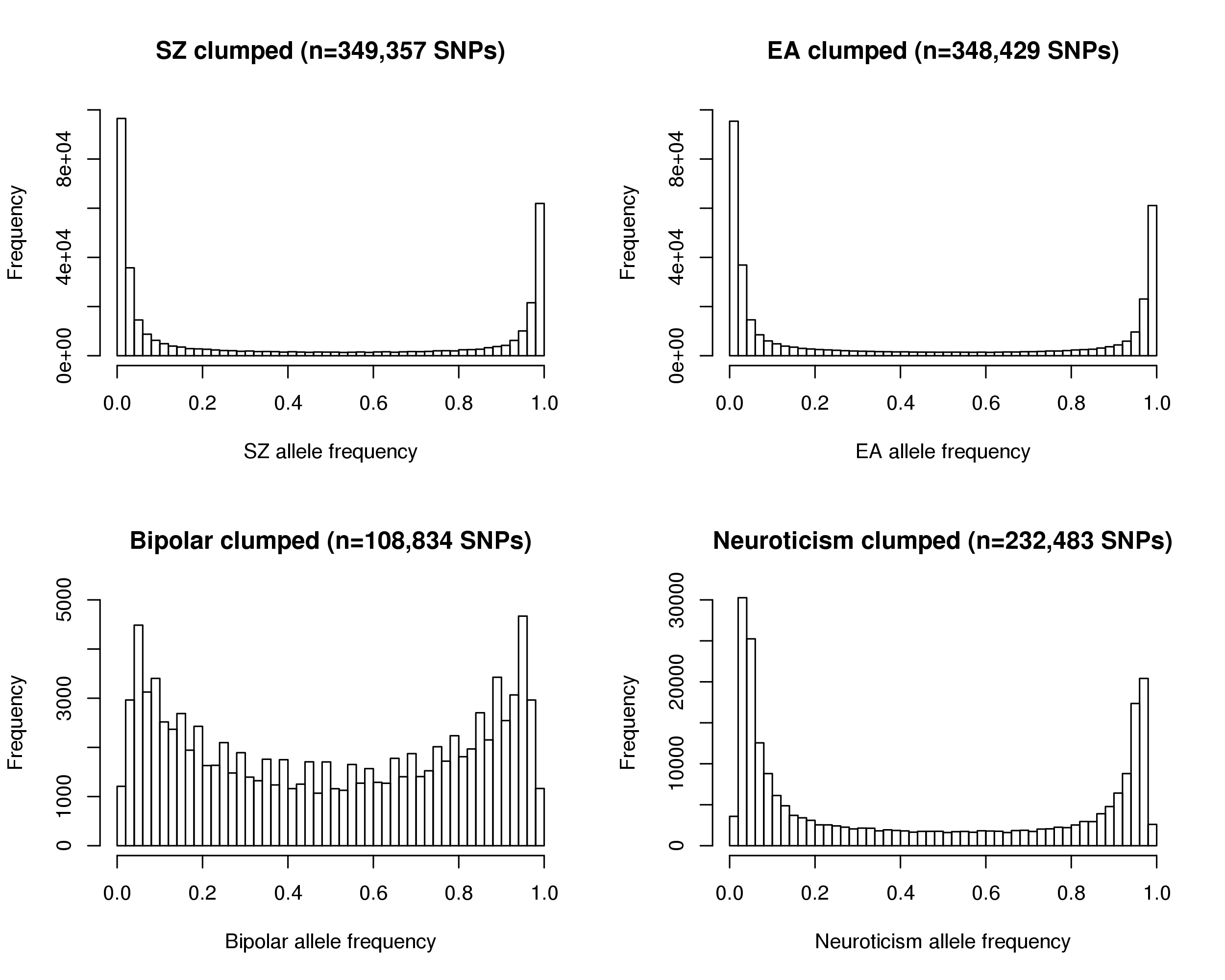


*Notes:* Histogram showing the allele frequency distribution for the lead SNPs used for polygenic prediction.

Supplementary Figure 7: Phenotypic correlations in the GRAS data collection among SZ cases


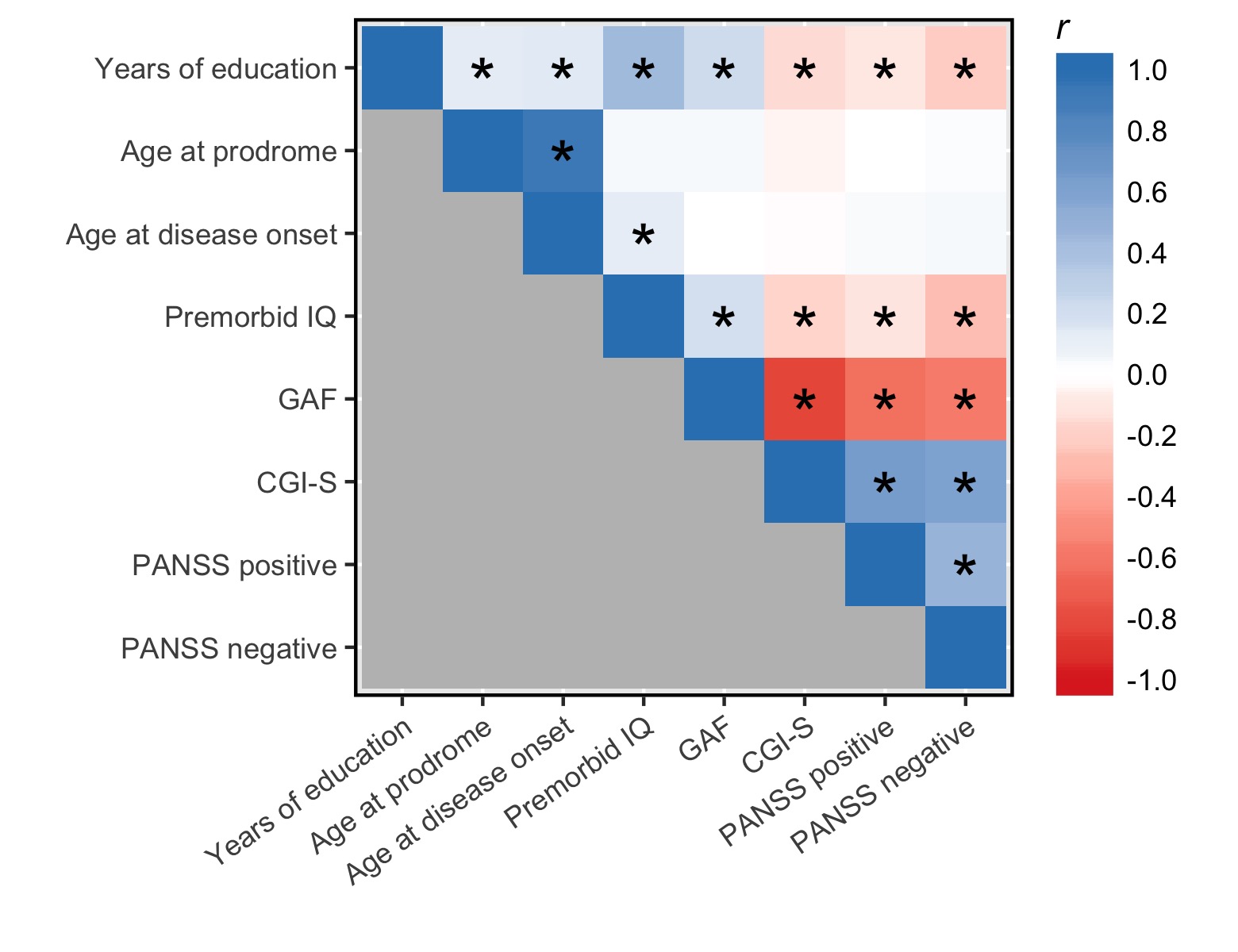


*Notes:* Phenotypic correlations (Pearson’s *r*) among schizophrenia patients in the GRAS data collection. The direction of the correlations is indicated by the coloring (blue for positive, red for negative) and the magnitude of the correlations is indicated by the gradient of the color. Black dots indicate nominal *P* < 0.01. GAF: Global Assessment of Functioning. CGI-S: Clinical Global Impression of Severity. PANSS: Positive and Negative Syndrome Scale. See detailed statistics in **Supplementary Data 15.** and detailed description of the measures in **Supplementary Note 5**.

Supplementary Figure 8: Q–Q plot of the 346 EA-associated SNPs for “unique” SZ_(min BIP)_.


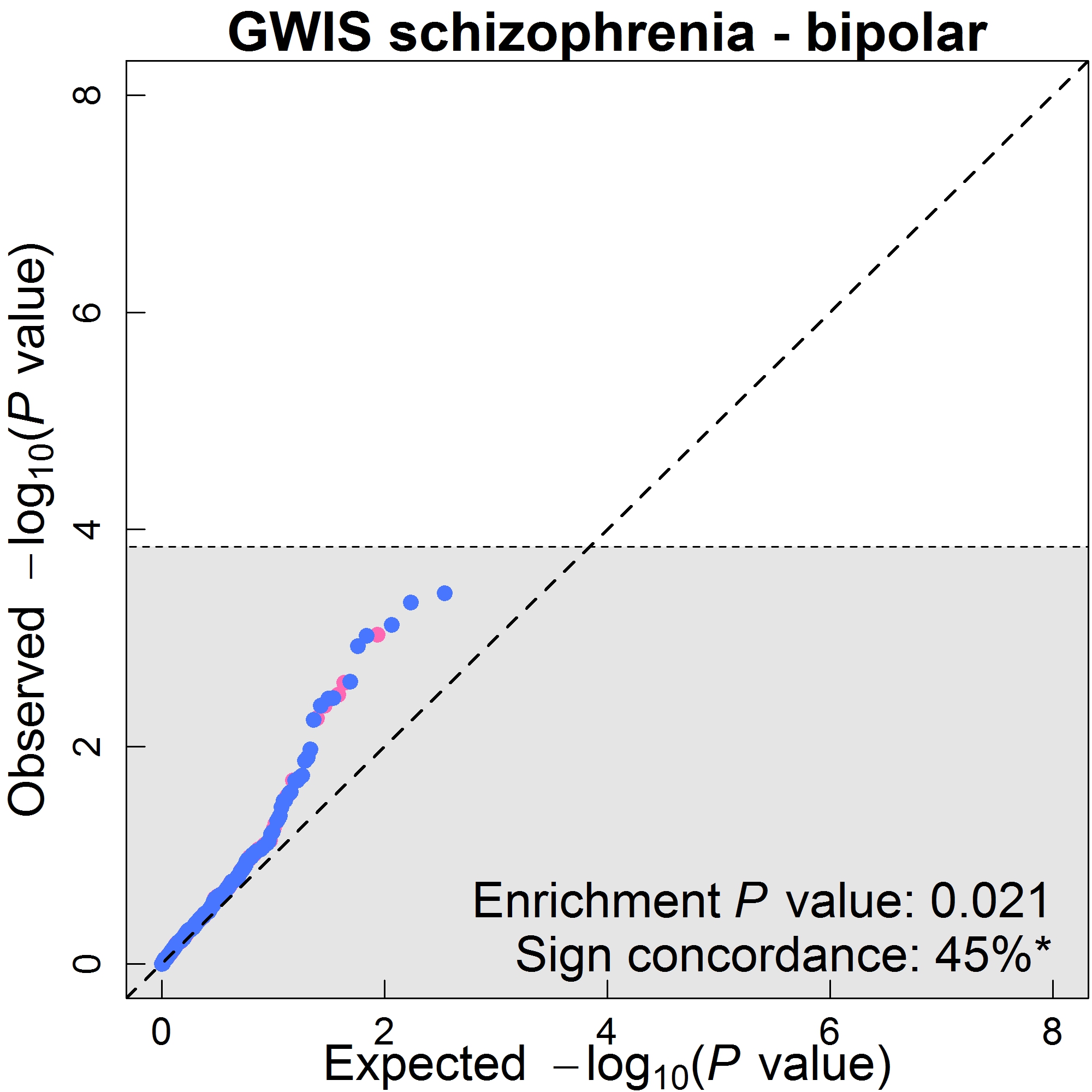


*Notes:* SNPs with concordant effects on both phenotypes are pink, and SNPs with discordant effects are blue. SNPs outside the grey area would have passed the Bonferroni-corrected significance threshold that corrects for the total number of SNPs we tested (*P* < 0.05/346 = 1.445×10^-4^). Observed and expected *P* values are on a −log_10_ scale. For the sign concordance test: *P* = 0.046, 2-sided.

Supplementary Figure 9: Results of the assortative mating simulations – raw enrichment *Z*-scores of causal SNPs for EA on SZ


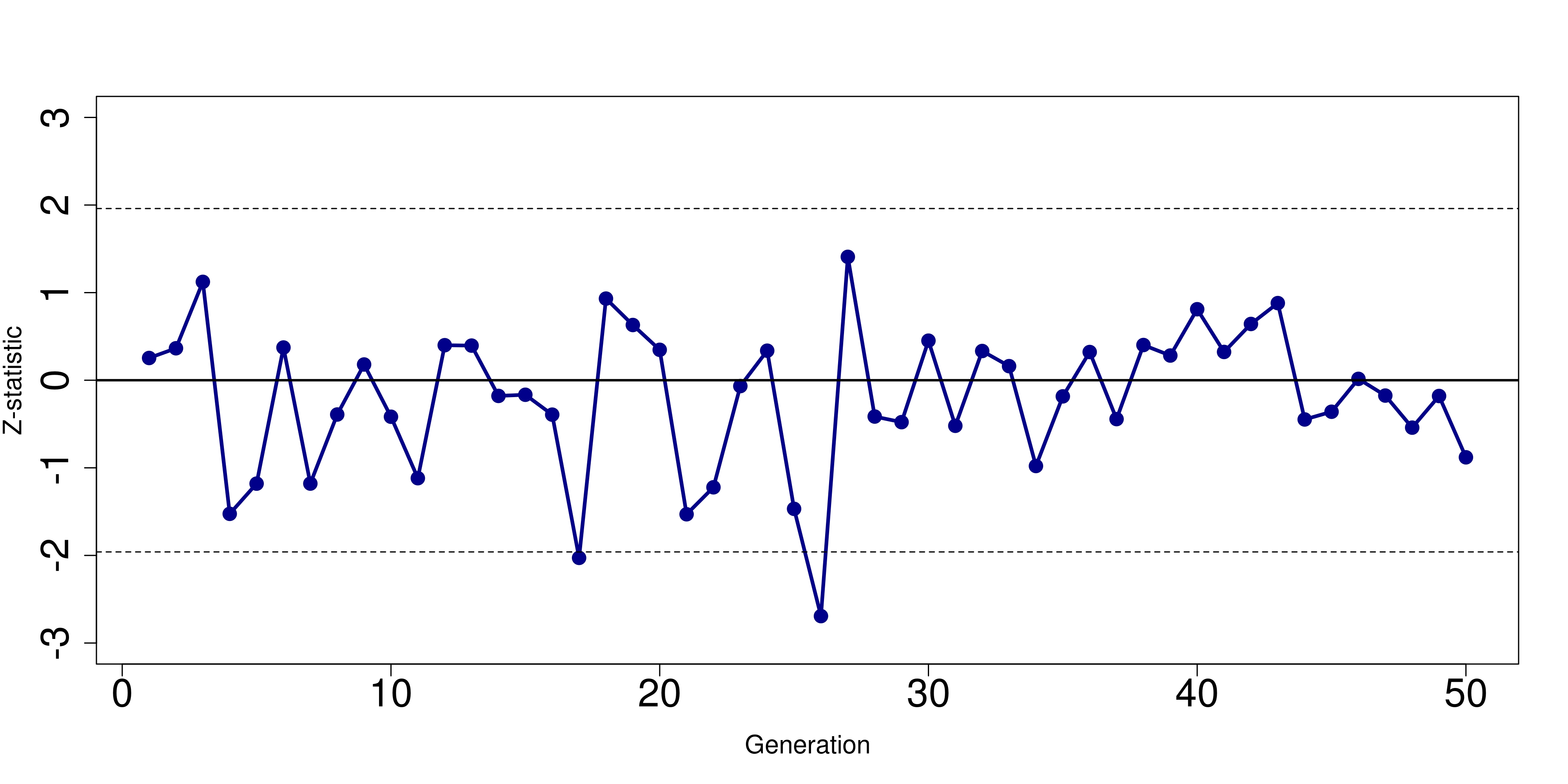


*Notes:* The graph shows the *Z*-scores of the Wilcoxon rank sum test (i.e. the raw enrichment *P* value) for each simulated generation. Causal genetic markers for EA and non-causal markers are tested for association with SZ. *Z*-score of $\pm1.96$ is shown by dashed lines.

# Statistical consideration

Genetic dependence under homogeneity

The concept of genetic correlation is frequently used in the field of quantitative genetics and is typically defined as the correlation of the true effects of single nucleotide polymorphisms (SNPs) on two outcomes^1,2^. Although the true effects of SNPs are unknown, estimates of genetic correlation can be obtained from the noisy estimates of SNP effects obtained from GWAS^1^ or they can be inferred from the phenotypic similarity of individuals conditional on their (estimated) genetic similarity^3^.

The broader concept of genetic dependence is, however, less well understood. In fact, for polygenic traits, a convincing definition of genetic dependence that resonates both with intuition and with the definition of dependence between random variables in probability theory is difficult to formulate. Here, we define genetic dependence in a manner that genetic dependence does not necessarily imply genetic correlation and, *vice versa*, that genetic correlation does not necessarily imply genetic dependence.

More specifically, given two traits, *A* and *B*, with proportion π*_A_* (resp. π*_B_*) of independent variants associated with *A* (*B*), and π*_AB_* associated with both traits, we define genetic dependence as π*_AB_* > π*_A_*π*_B_*. Simply put, under this definition, two traits are considered genetically dependent in case they share more causal (or causal-loci tagging) variants than expected by chance. As a result, π*_AB_* = π*_A_*π*_B_* implies independence. Finally, π*_AB_* < π*_A_*π*_B_* is also a form of dependence, where an increase in probability of a variant being associated with *A*, decreases the chance of that variant also being also associated with *B*.

This definition of genetic dependence, however, says very little about how the genetic value of *A* influences the distribution of the genetic value of *B*. Consider, for example, the case where *A* and *B* are both highly heritable and genetically dependent (i.e., π*_AB_* > π*_A_*π*_B_*) and where the effects of the shared loci follow a random-effects model, but in which the effects of these shared loci are not correlated across traits. In this scenario, the genetic correlation between these traits is zero. Nevertheless, an individual for whom *A* takes on a rather extreme value is also likely to have an extreme value for *B*, yet we do not know anything about the direction in which *B* will go given *A*.

This example reveals that true independence, as it is defined in probability theory, may be a rare event in the context of complex trait genetics. Moreover, under our definition of independence (i.e., π*_AB_* = π*_A_*π*_B_*), traits *A* and *B* can still be genetically correlated, *viz*., when the effects of the shared loci are correlated across traits. Hence, under our definition, genetic dependence is not equal to genetic correlation; genetic correlation does not imply dependence, and genetic dependence does not imply correlation.

Heterogeneity

Up until this point, we have implicitly considered genetically homogeneous traits, where a subset of variants is shared among traits, and the effects of these shared variants have a uniform correlation across traits. However, a trait can be genetically heterogeneous in the sense that it aggregates over several endophenotypes or symptoms that are not genetically identical (i.e. their genetic correlation is not 1). One can imagine a scenario where several endophenotypes are affecting traits *A* and *B*, where some of the endophenotypes affect both traits positively or both negatively, some endophenotypes affect one trait positively and the other trait negatively, and other endophenotypes affect only either of the two traits. For example, consider the following structural model for *A* and *B*:

$$A= G_{1}+G_{2}+G_{3}+E_{a},$$

$$B=G_{1}-G_{2}+G_{4}+E_{B},$$

where *G*_1_ and *G*_2_ are endophenotypes affecting both traits, with *G*_1_ affecting *A* and *B* sign-concordantly and *G*_2_ affecting *A* and *B* with a different sign, *G*_3_ and *G*_4_ are endophenotypes both affecting only one trait, and *E* denotes the environmental component of a given trait, denoted by its subscript.

Obviously, when the variants involved in the four endophenotypes are not overlapping, the total effects of the variants shaping *G*_1_ on *A* and *B* are positively correlated at +1, whereas the effects of variants shaping *G*_2_ on *A* and *B* are negatively correlated at −1. Moreover, if Var(*G*_1_) = Var(*G*_2_) the genetic correlation of *A* and *B* will be zero, even when a disproportionate amount of variants shape *G*_1_ and *G*_2_ (i.e., disproportionate in the sense that π*_AB_* > π*_A_*π*_B_*).

Hence, we have devised a simple model where two traits are highly genetically dependent (i.e., π*_AB_* > π*_A_*π*_B_*) and have strongly correlated effects for subsets of shared loci, whilst the total genetic correlation between the traits is zero.

Genetic dependence of educational attainment and schizophrenia

In the particular case of schizophrenia (SZ) and educational attainment (EA), a small, positive genetic correlation ($\rho_{EA,SZ}\approx0.08$)^1,2^ and genetic dependence between both traits have been previously reported^2,4^. In particular, given that a variant is robustly associated with either trait, it significantly increases the chances of that variant also being associated with the other trait.

To illustrate this, **Supplementary Fig. 1** shows a scatter plot of all *Z*-statistics from the GWASs on EA and SZ in non-overlapping samples. As can be seen, there is inflation in the test statistics along both the horizontal and the vertical axes, highlighting variants that are likely to be associated with one of the two traits. However, there are also variants that reach marginal nominal genome-wide significance for both traits on both diagonal axes. Our tests using the raw enrichment *P* value formally show that SNPs associated with EA are more likely to be associated with SZ than expected by chance, given the overall distribution of *Z*-statistics for SZ. Yet, the sign of association with one trait is not very informative of the sign of association with the other trait. It seems, though, as if there are more SNPs with the same sign for both traits that reach marginal significance than there are SNPs that have discordant signs. This is consistent with the idea that both traits are genetically dependent (i.e., more shared loci than expected by chance) with a subtle positive genetic correlation.

However, clearly there are also outliers which seem to defy expectations under a positive genetic correlation between both traits, *i.e.,* we see SNPs that are robustly associated with both traits, but with opposing signs in the effect estimates.

Nevertheless, if we assume for now that both traits do actually have a homogenous co-architecture, then the ‘effective genetic correlation’ (i.e., correlation as inferred from genome-wide data; *ρ*_G_) is expected to be given by the product of π*_AB_* and the correlation in the effects of shared loci (denoted by *ρ*), *ρ*_G_ = *ρ*π*_AB_*^5^.

However, even when *ρ* is large (e.g., *ρ* = 1) and when both π*_A_* and π*_B_* are relatively small (e.g., π*_A_* = π*_B_* = 10%), it holds that a relatively small π*_AB_* (e.g., π*_AB_* = 2%) may still be in excess of π*_A_*π*_B_*, hence, constituting genetic dependence, whilst the effective genetic correlation remains small (e.g., *ρ*_G_ = 0.02). Therefore, such a simple model with a homogeneous co-architecture could easily explain two of our salient empirical results, *viz*., genetic dependence (i.e. the raw enrichment *P* value) in conjunction with low genetic correlation as inferred from genome-wide data^1,2^.

Yet, there are two additional stylized results from our empirical analyses below that seem difficult to reconcile with a simple, linear relationship between SZ and EA. First, we observe that by decomposing a polygenic score (PGS) for SZ into two scores, one based on variants of which the effect estimates are sign-concordant with EA and another based on variants for which the sign of the effect estimates is dis-concordant across traits, hardly boosts the predictive accuracy for SZ itself (**Supplementary Data 14**). Second, this split-PGS approach does increase the predictive accuracy for the symptoms of SZ (**Table 2** and **Supplementary Data 18**, **20**, **21**, and **22**).

Simulation study

In order to explain these two additional stylized results, we will now consider several simulations to get a better understanding of what type of data-generating process may explain these salient features. We do so by considering a GWAS sample of 25k individuals and a hold-out sample for prediction of 5k individuals. We draw 25k independent biallelic SNPs in Hardy-Weinberg equilibrium, with allele frequencies uniformly distributed between 5% and 95%. In each simulation, we simulate two phenotypes, *A* and *B*, with 2.5k causal variants per trait, shaping a trait heritability of 50% for both.

The proportion of overlapping SNPs is considered at three levels, *viz*., 0%, 1%, and 10%. The correlation in the effects of overlapping SNPs is considered at five levels, *viz*., −1, −0.5, 0, +0.5, and +1. Hence, in each of these simulations the traits have a homogeneous co-architecture (i.e., consistently correlated effects for variants associated with both traits).

However, we also consider a simulation where the true correlation in the effect of overlapping SNPs is a mix of −1 for 40% of the shared SNPs, +1 for an additional 40% of the shared SNPs, and zero for the remaining 20% of the shared SNPs. Such a pattern could arise under a scenario where there are several endophenotypes involved, one affecting both traits positively, one affecting both traits negatively, one affecting only *A*, and one affecting only *B*. Importantly, at the genome-wide level these negative and positive correlations cancel out by aggregation, yielding an overall genetic correlation equal to zero.

Summarizing, we have six levels for correlations in the effects (i.e., −1, −0.5, 0, +0.5, +1, and a mixture) and three levels for the proportion of shared SNPs. This means we have 18 distinct designs in total.

For each design, we run one simulation to infer *Z*-statistics of GWASs for both simulated traits. Scatter plots of these *Z*-statistics for traits *A* and *B* are reported per simulation design in subplots contained in **Supplementary Fig. 2**. We perform 100 additional simulations per design, to infer the average difference in *R*^2^ in a model explaining *A* in the hold-out sample using only the PGS for *A* and the PGS for *B*, and a model where *A* is explained using the PGS for *B* and the split PGS for *A*. For each design, this average incremental *R*^2^ is reported above the corresponding subplot in **Supplementary Fig. 2**.

As **Supplementary Fig. 2** demonstrates, the closest we can get to the empirical distribution of *Z*-statistics (shown in **Supplementary** **Fig. 1**) is by a combination of at least 1% of loci being shared (two rightmost columns) and a positive genetic correlation (two penultimate rows), as well as a more extreme scenario, where there are shared loci (two rightmost columns) with mixed correlations across traits (last row).

In case of a homogeneous co-architecture of the two traits (i.e., any design other than what is reported in the last row of **Supplementary Fig. 2**), decomposing a PGS for *A* into two scores, one based on variants of which the effect estimates are sign-concordant across traits and another based on variants for which the sign of the effect estimates is dis-concordant across traits, only helps to boost predictive accuracy for *A* when the proportion of shared loci is larger than what is anticipated by chance (last column) and when the correlation in the SNP effects across traits is different from zero (first two rows and penultimate two rows). The reason for this boost is likely to be the fact that, in case of a positive (resp. negative) genetic correlation, sign-concordant (dis-concordant) scores are better at teasing out the variants that matter for both traits and are, therefore, able to use that information to yield a predictive advantage.

However, under an effective genetic correlation between SZ and EA close to zero (as we observe it to be empirically), our simulations do not reveal a boost in predictive accuracy for SZ itself by splitting its PGS by sign-concordance. Moreover, if symptoms of SZ are merely a function of the disease itself and some additional environmental factors, then seeing a boost in predictive accuracy of symptoms by concordance and dis-concordance PGSs is even more far-fetched.

By considering a heterogeneous case, however, all stylized findings (i.e., (i) a low genetic correlation between *A* and *B* at a genome-wide level, in spite of (ii) significant enrichment for associated loci, (iii) hardly any boost in predictive accuracy for *A* by splitting the PGS, and yet (iv) a substantial boost in prediction of symptoms of *A* by splitting the score) can be satisfied at the same time.

We show this by extending the simulation design reported in the lower right corner of **Supplementary Fig. 2**, where the proportion of SNPs shared between *A* and *B* is 10% and where the correlation in the effects of overlapping being mixed (as explained before). We assume that there are three symptoms: Symptom 1, which is shaped by an endophenotype that affects both *A* and *B* positively (i.e., it comprises the SNPs that have positively correlated effects across traits); Symptom 2, which is shaped by an endophenotype that affects *A* positively and *B* negatively (i.e., it comprises the SNPs that have negatively correlated effects across traits); Symptom 3, which is shaped by an endophenotype that only affects *A* (i.e., it comprises SNPs that only affect *A*, and shared SNPs of which the effects are not correlated across traits). Hence, Symptoms 1 and 2 correspond to endophenotypes affecting both *A* and *B* but in a heterogeneous manner, whereas Symptom 3 corresponds to an endophenotype which has nothing to do with *B*. We predict, therefore, that exploiting information from *B* will only improve predictive accuracy for Symptoms 1 and 2 significantly. Moreover, in light of the heterogeneity, we predict that splitting the PGS for *A* by concordance with *B* will further boost the predictive accuracy of Symptoms 1 and 2.

**Supplementary Data 1** reports the *R*^2^ of several PGS-based models aimed at explaining the symptoms of *A* in a hold-out sample. For Symptoms 1 and 2, which are effectively functions of different endophenotypes, the predictive accuracy is indeed boosted by including information from both traits. In addition, as expected, the model where the PGS for *A* is split according to sign-concordance with the GWAS estimates for *B* (also including PGS for *B* as regressor) outperforms the model only including the whole PGS for *A* and the PGS for *B*; by doing so the predictive accuracy is boosted by about 1% for both symptoms. On the other hand, for Symptom 3, a function of endophenotypes that are unrelated to Trait *B*, nothing is gained by including information about *B*.

**Conclusions**

We have considered a wide range of simulations for two genetically dependent and/or correlated traits. In case of genetically correlated traits, this correlation was shaped by a set of overlapping SNPs, with a homogenous correlation in the effects of those SNPs across traits (i.e., a homogeneous co-architecture). For dependence, we considered shared loci in excess of what is expected by chance, in combination with both consistent correlations in the effects of variants across traits as well as correlations being mixed, depending on which endophenotypes they pertain to.

The best explanation for the salient features of our empirical results (i.e., low genetic correlation across traits in spite of strong genetic dependence, hardly any boost in predictive accuracy of SZ by splitting its PGS by sign-concordance with EA, and yet a strong boost in predictive accuracy of the symptoms of SZ by splitting its PGS) is found in a scenario where (i) the amount of shared causal loci is in excess of expectation, (ii) with these loci tagging different endophenotypes, and (iii) where the effects of these endophenotypes differ in sign across traits and symptoms.

Hence, the most reasonable explanation for these findings is genetic heterogeneity in either or both traits. It is important to note here that, even though (i) the specific data-generating processes we considered help to effectively rule out the simplest structural model (i.e., where *A* and *B* have a homogeneous co-architecture) and (ii) our heterogenous model comprising only a handful of endophenotypes can already explain important features of our results, these findings do not bar the possibility that the true nature of these traits may be far more complex than the small set of endophenotypes, with both concordant and dis-concordant signs across traits and symptoms, that we have considered.

Improved polygenic prediction of endophenotypes and symptoms

In this section, we develop a simple statistical test for genetic heterogeneity in a phenotype. We start by explaining why and how polygenic scores from various genetically correlated traits may help to increase predictive accuracy. Then, we extend these thoughts to predict endophenotypes or symptoms of a trait for which large-scale GWAS results may still be missing. Finally, we present a simple test for genetic heterogeneity among these endophenotypes or symptoms that is based on a combination of GWAS summary statistics from two genetically dependent traits that are available.

Improved polygenic prediction with GWAS results from genetically correlated traits

Genetic prediction with polygenic scores (PGSs) of an outcome, $y$, can often be improved upon by adding PGSs of related traits, e.g., $z$. The extent of this improvement depends on the genetic correlation between the two traits as well as the size and independence of the measurement errors in PGSs.

To show this mathematically, we consider the following model:

$$y=\gamma S_{y}^{*}+\epsilon$$

where $y$ is the outcome, $S_{y}^{*}$ is the *true* PGS (constructed from a GWAS on $y$ in an infinitely large sample of the target population) with effect size $\gamma$, and $\epsilon$ is an error term which includes environmental effects. For convenience, we assume that $y$ is standardized to a mean of 0 and a variance of 1. We assume the available PGS ($S_{y}$) is observed with measurement error ($e_{y}$) that comes from GWAS analyses in finite sample sizes,

$S_{y}=S_{y}^{*}+e_{y}$. For convenience, we assume that the measured PGS is also standardized. We will study the difference in $R^{2}$ between only using $S_{y}$ as a predictor and adding a PGS of a genetically correlated trait, $S_{Z}$, as a second predictor.

Similarly, the available PGS for a related trait $z$ is

$$S_{z}=S_{z}^{*}+e_{z}.$$

The residual of a regression of $y$ on $S_{y}$ and $S_{Z}$, $\tilde{\epsilon}$, can be written as:

$$\tilde{\epsilon}=M_{{S_{Z}S}_{y}}y=M_{S_{Z}}M_{S_{y}}y=M_{S_{Z}}\hat{\epsilon}$$

where $M_{{S_{Z}S}_{y}}$ is an orthogonal complement matrix to a projection on $S_{y}$ and $S_{Z}$, $M_{S_{Z}}$ and $M_{S_{y}}$, are the orthogonal complement matrices to a projection on $S_{Z}$ and $S_{y}$ respectively, and $\hat{\epsilon}$ is the residual of a regression of $y$ on only $S_{y}$. This shows that the residual of regressing $y$ on $S_{y}$ and $S_{Z}$ is equal to the residual of regressing $\hat{\epsilon}$ on $S_{Z}$, such that this residual is smaller or equal to $\hat{\epsilon}$. Furthermore, this will simplify derivations.

We rewrite $y$ in terms of its measured score, $S_{y}$:

$$y=\gamma S_{y}^{*}+\epsilon= \gamma S_{y}-\gamma e_{y}+\epsilon$$

The expected estimated coefficient $\gamma$ is equal to:

$$E\left[ \hat{\gamma} \right]=\frac{cov(S_{y}, y)}{var(S_{y})}= \gamma var(S_{y}^{*})$$

The estimated coefficient $\hat{\gamma}$ is biased due to the measurement error in $S_{y}$. The size of the bias depends on the size of the variance of $S_{y}^{*}$ relative to the variance of $S_{y}$ (which is standardised to equal 1). In other words, it depends on how much of the variance of the PGS can be attributed to the variance of the true genetic effects. Note that since the variance of $S_{y}^{*}$ is smaller than 1, the bias of $\hat{\gamma}$ will be towards 0. De Vlaming et al. derive the size of this bias, which primarily depends on GWAS sample size^5^.

The residual of this regression is equal to:

$$\hat{\epsilon}=y- \hat{\gamma}S_{y}=\gamma S_{y}-\gamma e_{y}+\epsilon-\hat{\gamma}S_{y}=\left( \gamma-\hat{\gamma} \right)S_{y}^{*}-\hat{\gamma}e_{y}+\epsilon$$

The residual of a regression of $y$ on $S_{y}$ and $S_{Z}$ is equivalent to the residual of a regression of $\hat{\epsilon}$ on $S_{Z}$, as can be seen from the earlier orthogonal projections.

$$\hat{\epsilon}=\delta S_{Z}+\epsilon^{o}$$

The expected estimated coefficient of regressing $\hat{\epsilon}$ on $S_{Z}$ is equal to:

$$E\left[ \hat{\delta} \right]=E[\frac{cov\left( \hat{\epsilon},S_{Z} \right)}{var\left( S_{Z} \right)}]= E\left[ cov\left( \left( \gamma-\hat{\gamma} \right)S_{y}^{*}-\hat{\gamma}e_{y}+\epsilon,S_{Z} \right) \right]=(\gamma-E[\hat{\gamma}]) cov\left( S_{y}^{*} ,S_{Z}^{*} \right)-E[\hat{\gamma}]cov(e_{y},e_{z})$$

The expectation of this coefficient can be split in two parts. First, $(\gamma-E[\hat{\gamma}]) cov\left( S_{y}^{*} ,S_{Z}^{*} \right)$: this is how much the second PGS will explain due to a underestimation of $\hat{\gamma}$ (which is $\gamma-E[\hat{\gamma}]$). The size of this first part depends on the size of the bias and $cov\left( S_{y}^{*} ,S_{Z}^{*} \right)$, which is directly related to how much the two traits are genetically correlated. The sign of the first part will equal the sign of $\gamma$ as $\hat{\gamma}$ is biased towards 0 and $cov\left( S_{y}^{*} ,S_{Z}^{*} \right)$ will always be positive.

The second part, $-E[\hat{\gamma}]cov(e_{y},e_{z})$, is a reduction of the explanatory power of the second PGS $S_{Z}$ in case it has partly the same measurement error (e.g. if the GWAS on $y$ and $z$ were carried out in partly overlapping samples). This second part will have the opposite sign of $\gamma$ due to the minus sign in front of the term ($E[\hat{\gamma}]$ has the same sign as $\gamma$ and $cov(e_{y},e_{z})$ is always positive).

The residual of this second regression is equal to:

$$\tilde{\epsilon}=\hat{\epsilon}-\hat{\delta}S_{Z}$$

Now we can calculate the difference in the estimated $R^{2}$ of a regression of $y$ on $S_{y}$ excluding and including $S_{Z}^{*}$:

$$R_{S_{y,}S_{Z}}^{2}-R_{S_{y}}^{2}=\left( 1-\frac{\sum_{i=1}^{N} {\tilde{\epsilon_{i}}}^{2}}{\sum_{i=1}^{N} y_{i}^{2}} \right)-\left( 1-\frac{\sum_{i=1}^{N} {\hat{\epsilon}_{i}}^{2}}{\sum_{i=1}^{N} y_{i}^{2}} \right)= \frac{\sum_{i=1}^{N} {(\hat{\delta}S_{Z})}^{2}}{\sum_{i=1}^{N} y_{i}^{2}}$$

which is generally greater than zero. Thus, we have shown that a polygenic score from an “off-target” phenotype that is genetically correlated with the target trait can help to improve predictive accuracy. The increase of $R^{2}$ will depend on the size of $\hat{\delta}$.

Detecting genetic heterogeneity among endophenotypes or symptoms

We move on to consider the polygenic prediction of a genetically heterogeneous outcome, i.e. a phenotype that can be decomposed into endophenotypes or symptoms of non-identical genetic architectures. Extending the logic from above, it is clear that these endophenotypes or symptoms can be predicted by polygenic scores to some extent even if GWAS results on the endophenotypes or symptoms are not directly available. The extent to which this will be possible depends on the genetic correlation between each symptom and the phenotypes *y* and *z* for which GWAS results are available.

Furthermore, since a PGS is a sum of linear effects, we can consider splitting the PGS into several scores. These scores can then also be used to predict the disease and its symptoms. If the disease and its symptoms are genetically homogenous, splitting the PGS into several scores should not add any predictive accuracy. However, if there is genetic heterogeneity in the symptoms, splitting the score by some informative criterion that is related to these symptoms may increase predictive accuracy. This additional information can, for instance, be gathered from GWAS results of a genetically dependent trait that was studied in an independent GWAS sample. One simple way to incorporate this information is by splitting SNPs depending on their sign concordance with both traits.

To show this mathematically, consider a model where an outcome, $y$, is a function of a score of *true* polygenic effects, $S^{*}$, and an error term with environmental effects, $\epsilon$.

$$y=\gamma S^{*}+\epsilon$$

The PGS is a sum of the effects of all markers, $x_{j}$, belonging to a set of markers, $X$, with corresponding *true* effect size $\beta_{j}^{*}$.

$$S^{*}=\sum_{x_{j}\in X} \beta_{j}^{*}x_{j}$$

The observed PGS, $S$, is constructed using estimated effects, $\hat{\beta}_{j}$, which includes estimation error $e_{j}$. More specifically,

$$S=\sum_{x_{j}\in X} \hat{\beta}_{j}x_{j},\mathrm{where}$$

$$\hat{\beta}_{j}=\beta_{j}^{*}+e_{j}.$$

The score, $S$, can be used to predict the outcome, $y$. The estimated coefficient for $S$, will be attenuated due to measurement error in the score.

Assume we have several outcomes, $z_{i}$, that have a linear dependence on $y$. By substitution, this dependence can be rewritten as follows:

$$z_{i}={\alpha_{i}+\delta}_{i}y+\eta_{i}=\alpha_{i}+\delta_{i}\gamma S^{*}+ \eta_{i}+\delta_{i}\epsilon,$$

where $\delta_{i}$ is the effect size of $y$ on $z_{i}$ and $\eta_{i}$ is an error term. The coefficients $\delta_{i}\gamma$ can be rewritten as a new coefficient $\theta_{i}$ and the error terms $\eta_{i}+\delta_{i}\epsilon$ can be rewritten as error $u_{i}$. That is, the model can be rewritten as:

$$z_{i}={\alpha_{i}+\theta}_{i}S^{*}+u_{i}.$$

Again, we can use the measured PGS to predict $z_{i}$. The estimated coefficient, $\hat{\theta}_{i}$, will be attenuated due to measurement error in $S$ with respect to the true score, $S^{*}$.

Since the PGS is a sum of effects of all markers, we can divide the score into two parts, by partitioning the set of markers, $X$, into two subsets, $X_{1}$ and $X_{2}$, to create two PGSs, $S_{1}^{*}$ and $S_{2}^{*}$.

$$S_{1}^{*}=\sum_{x_{j}\in X_{1}} \beta_{j}^{*}x_{j}$$

$$S_{2}^{*}=\sum_{x_{j}\in X_{2}} \beta_{j}^{*}x_{j}$$

The true PGS is then equal to the two scores added together, and we can write both $y$and $z_{i}$ as a function of $S_{1}^{*}$ and $S_{2}^{*}$.

$$S^{*}=S_{1}^{*}+S_{2}^{*}$$

$$y=\gamma S^{*}+\epsilon=\gamma{(S}_{1}^{*}+S_{2}^{*})+\epsilon$$

$$z_{i}=\alpha_{i}+\theta_{1,i}S_{1}^{*}+\theta_{2,i}S_{2}^{*}+v_{i}$$

We can estimate $\theta_{1,i}$ and$\theta_{2,i}$ by replacing $S_{1}^{*}$ (resp. $S_{2}^{*}$) by $S_{1}$ ($S_{2})$.Again the coefficients will suffer from an attenuation bias. If we assume that the measurement error is proportional to the variance on $S_{1}^{*}$ and $S_{2}^{*}$^[[1]](#footnote-1)^, then the attenuation bias in both $\hat{\theta}_{1,i}$ and$\hat{\theta}_{2,i}$ will be equal. If, in addition, $y$ and $z_{i}$ are genetically homogenous, $\hat{\theta}_{1,i}$ and$\hat{\theta}_{2,i}$ should not differ significantly.

The model with a single PGS is nested in the model with the PGS split into two or more components, as the model with the single PGS implies that the coefficients for the two components of the PGS are equal to each other. To see this, first note that $X_{1}$ and $X_{2}$ are a partition of $X$. Hence, we can split the total PGS as follows:

$$S=\sum_{x_{j}\in X_{1}} \hat{\beta}_{j}x_{j}+\sum_{x_{j}\in X_{2}} \hat{\beta}_{j}x_{j}=S_{1}+S_{2}.$$

Also note that the regression of $z_{i}$ on $S$ can be formulated in terms of the following model:

$$z_{i}=\alpha_{i}+\lambda_{i}S+v_{i}.$$

Finally, note that the regression of $z_{i}$ on $S_{1}$ and $S_{2}$ can be written in terms of the model below:

$$z_{i}=\alpha_{i}+\lambda_{1,i}S_{1}+\lambda_{2,i}S_{2}+v_{i}.$$

This last model can rewritten as follows:

$$z_{i}=\alpha_{i}+\lambda_{1,i}(S_{1}+S_{2})+{(\lambda}_{2,i}-\lambda_{1,i})S_{2}+v_{i}$$

$$=\alpha_{i}+\lambda_{1,i}S+{(\lambda}_{2,i}-\lambda_{1,i})S_{2}$$

$$=\alpha_{i}+\lambda_{i}S+\pi_{i}S_{2}+v_{i}.$$

From this last expression for $z_{i}$ it follows that regression of $z_{i}$ on $S_{1}$ and $S_{2}$ is identical to a regression of $z_{i}$ on $S$ and $S_{2}$, in the sense that the subspace spanned by $S_{1}$ and $S_{2}$ is identical to the subspace spanned by $S$ and $S_{2}$. Ergo, the regression of $z_{i}$ on $S_{1}$ and $S_{2}$ is nested with respect to the regression of $z_{i}$ on $S$, in terms of the subspaces being spanned by respective sets of regressors, enabling us to formally test the hypothesis of genetic homogeneity with an *F*-test.

If we can reject this hypothesis, this means that the original model with a single PGS was misspecified, i.e., the effects of the genetic variants, $x_{j}$, on $z_{i}$ are not all mediated through a single variable $y$.

In our particular case, splitting the PGS for SZ into several components should not help to predict symptoms unless these symptoms are genetically heterogeneous. In other words, if we find that splitting the PGS for SZ based on the sign concordance of SNPs with EA improves predictive accuracy of disease symptoms or severity, or if the coefficients for the *Concordant* and *Discordant* scores are significantly different from each other, this would be evidence for genetic heterogeneity in SZ. We present the results of such tests in **Table 2** and **Supplementary Data 18**, **20**, **21**, and **22**.

# Selection of education-associated candidate SNPs

Ideally, proxy-phenotype analyses should use a pre-specified *P* value threshold that maximises the expected number of true positive results in the look-up stage. The optimal threshold trades off between two opposing effects. On the one hand, a less stringent threshold yields a larger number of candidates that are forwarded to the second stage. A larger set of candidates is more likely to contain true positives. On the other hand, a larger number of candidates requires that a more stringent experiment-wide significance level needs to be applied in the second stage to adjust for multiple testing, which decreases power to pick out the true positives from among the set of candidates^6^. In principal, it is possible to calculate the optimal *P* value threshold based on the observed distribution of standardised effects sizes and standard errors in the GWAS results of the proxy, the genetic correlation between the two traits, their SNP-based heritabilities, and the sample size of the target phenotype. Given these parameters, one can infer the expected effect size of a genetic variant on the target from the results on the proxy phenotype, calculate the statistical power for the look-up, and approximate the number of expected true positive associations from the look-up at various *P* value thresholds^7^.

In the current case, the value of such theoretical calculations is limited because the genetic correlation between EA and SZ does not reflect the actual genetic overlap between the two traits adequately. Specifically, Okbay et al.^2^ report low, but significant, bivariate LD score regression estimates for the genetic correlation between EA and SZ of 0.08 (*P* = 3.2×10^-4^). In addition, the 74 genome-wide significant EA loci have only 51% sign concordance with the SZ results. This sign concordance pattern cannot be differentiated from what would be expected by chance for two traits that exhibit no genetic overlap. Yet, the same 74 EA-associated loci are strongly enriched for association with SZ (enrichment *P* value < 0.002), which strongly rejects the hypothesis of no genetic overlap between the two traits. This implies that standard formulas^7^ to calculate the expected effect size of a genetic variant on SZ from the observed results on EA will be too conservative because they ignore the specific pattern of split sign concordance but strong enrichment for this pair of traits.

Instead of calculating a noisy theoretical optimum, we follow Rietveld et al.^6^ and selected
10^-5^ as the default *P* value threshold prior to carrying out the proxy-phenotype analyses (<https://osf.io/dnhfk/>).

# Pleiotropy analyses with PAINTOR

We report the results of the pleiotropy analyses with PAINTOR for the broadest and narrowest sets we constructed (90% and 50%, respectively) in **Table 1** in the main manuscript and in **Supplementary Data 4**. **Supplementary Data 4** also contains the results for 80% and 65% credible sets. Furthermore, we report the correlation between SZ and EA *Z*-statistics for the 65% credible SNP sets (striking a balance between sets large enough to compute a sensible correlation, and not so large as to contain many SNPs with low probability of being causal) in **Supplementary Data 4** – these indicate the sign of the relation between SZ and EA at this particular locus.

For the broadest set analyses (90%), we found that eleven loci have a medium or high credibility to have direct causal effects on both EA and SZ (including one of the novel SNPs, rs7336518). Six of these loci have concordant effects on the two traits (i.e., ++ or --) while five have discordant effects (i.e., +- or -+, **Table 1** and **Supplementary Data 4**).

We included LocusZoom plots (**Supplementary Fig. 3**) that illustrate the three scenarios of interest: (1) a concordant SNP with high probability of pleiotropy (rs79210963) (**Supplementary Fig. 3a**), (2) a discordant SNP with medium probability of pleiotropy (rs7610856) (**Supplementary Fig. 3b**), and (3) a SNP with low probability of pleiotropy (rs11694989) (**Supplementary Fig. 3c**). In all three cases, the SNP of interest is the top SNP from the GWAS on EA and the plots illustrate the LD-structure of this SNP in both the EA and SZ GWAS results. The concordant SNP (rs79210963) (**Supplementary Fig. 3a**) is in high LD ($r_{LD}^{2}=0.96$ ) with the most strongly associated SNP for SZ in this region for which LD information was available (rs112509803). The same holds for the discordant SNP (rs7610856) (**Supplementary Fig. 3b**) which is in high LD ($r_{LD}^{2}=0.98$) with the top hit for SZ in this region (rs62244884), although the SNPs in high LD span across a number of genes in both cases. In contrast, the example SNP with low probability for pleiotropy (rs11694989) (**Supplementary Fig. 3c**) is the top hit for EA, but it is clearly not the top hit for SZ in that region (rs6704768). Indeed, the plot shows that the LD between rs11694989 and the top SZ in this region is low ($r_{LD}^{2}=0.27$ ). The region is also characterized by several short genes in close proximity to each other, suggesting that rs11694989 and the top hit for SZ in this region (rs6704768) could tag different genes.

One of the two novel candidate loci for SZ that our PPM analyses identified (rs7336518) has a medium likelihood of having a direct pleiotropic effect on both traits. Furthermore, SNPs in the surrounding area of rs7336518 have a perfect positive (i.e. concordant) correlation of *Z*-statistics for both traits.

The analyses of the 50% credible sets are based on a smaller number of SNPs. This also results in lower probabilities that the SZ set contains the causal SNP for EA and vice versa. Nevertheless, our analysis with 50% credible sets show that four specific loci (rs7610856, rs320700, rs79210963, and rs7336518) had credibility of more than 15% for the other trait, providing support for the high (rs320700 and rs79210963) and medium (rs7610856 and rs7336518) credibility judgments based on the 90% sets. One of these has a discordant effect (rs7610856) while the others have a concordant effect on SZ and EA.

We recognize that a second metric of importance in this analysis is the size of the credible set – a single SNP with a high probability of being a causal SNP for both traits (say 30%) may be stronger evidence of a shared a causal signal than a large set of SNPs (say several hundred) that have a higher probability (say 65%) of containing a causal locus for both traits. Specifically, the average number of SNPs in the 90% credibility sets were 438 and 290 for EA and SZ, respectively, with an average number of 2,765 SNPs per locus (defined as the 500Kb upstream and downstream genetic window included in the PAINTOR finemapping analysis). The narrower 50% credibility sets had on average 31 SNPs for EA and 20 for SZ (**Supplementary Data 4**). The size of the sets (in terms of number of SNPs) indicates the limits of statistical fine-mapping. Further progress would require a different approach such as cross-ethnic or experimental fine-mapping or analysis of sequence data in families.

# Biological annotation with DEPICT

**Significant reconstituted gene sets**

DEPICT identified 111 significant reconstituted gene sets at an FDR below 5% (**Supplementary Data 5**). To identify independent biological groupings, we computed the pairwise Pearson correlations of all significant gene sets using the “network_plot.py” script provided with DEPICT. Next, we used the Affinity Propagation method on the Pearson distance matrix to cluster the findings^8^. The Affinity Propagation method automatically chooses an exemplar for each cluster (**Supplementary Data 6**). **Supplementary** **Fig. 4a** visualises the results of this analyses, showing only one exemplar per gene sets (*n* = 19). We briefly describe the implicated gene sets below. The definitions are taken from AmiGO^9^, the Mouse Genome Database^10^, the Reactome pathway Knowledgebase^11^ and GeneCards^12^.

*npBAF complex* (7-set cluster) is named after the GO cellular component (GO:0071564) defined as “A SWI/SNF-type complex that is found in neural stem or progenitor cells”. The prefix *np* stands for *neural progenitor*. The npBAF complex is essential for the self-renewal/proliferative capacity of multipotent neural stem cells.

*Transcription cofactor activity* (12-set cluster) is named after the GO molecular function (GO:0003712) defined as “Interacting selectively and non-covalently with a regulatory transcription factor and also with the basal transcription machinery in order to modulate transcription. Cofactors generally do not bind the template nucleic acid, but rather mediate protein-protein interactions between regulatory transcription factors and the basal transcription machinery”.

*REACTOME_TRANSMISSION_ACROSS_CHEMICAL_SYNAPSES* (15-set cluster) is named after the Reactome pathway centred on genes involved in transmission across chemical synapses. Chemical synapses are specialised junctions that are used for communication between neurones, neurones and muscle or gland cells. The pre-synaptic neurone communicates via the release of neurotransmitter which binds the receptors on the post-synaptic cell.

*Dendrite* (21-set cluster) is named after a large and heterogeneous GO cellular component (GO: 0030425) defined as “A neuron projection that has a short, tapering, often branched, morphology, receives and integrates signals from other neurons or from sensory stimuli, and conducts a nerve impulse towards the axon or the cell body. In most neurones, the impulse is conveyed from dendrites to axon via the cell body, but in some types of unipolar neurone, the impulse does not travel via the cell body”.

*Abnormal cerebral cortex morphology* (5-set cluster) is named after the Mammalian Phenotype category (MP:0000788) defined as “any structural anomaly of a thin layer of grey matter on the surface of the cerebral hemisphere that folds into gyri”.

*Dendritic spine morphogenesis* (3-set cluster) is named after the GO biological process (GO:0060997) defined as “The process in which the anatomical structures of a dendritic spine are generated and organised. A dendritic spine is a protrusion from a dendrite and a specialised subcellular compartment involved in synaptic transmission”.

*REACTOME_AXON_GUIDANCE* (4-set cluster) is named after the Reactome pathway centred on genes involved in axon guidance. Axon guidance or axon pathfinding is the process by which neurones send out axons to reach the correct targets.

*GRIN2A PPI subnetwork* (9-set cluster) is named after the gene *GRIN2A* (glutamate ionotropic receptor NMDA-type subunit 2A). This gene encodes a member of the glutamate-gated ion channel protein family. The encoded protein is an N-methyl-D-aspartate (NMDA) receptor subunit. The most significantly enriched member set is “protein serine/threonine phosphatase complex”, which is named after GO cellular component (GO:0008287) defined as “A complex, normally consisting of a catalytic and a regulatory subunit, which catalyzes the removal of a phosphate group from a serine or threonine residue of a protein”.

*SNW1 PPI subnetwork* (3-set cluster) is named after the gene *SNW1* (SNW Domain Containing 1) encodes a coactivator that enhances transcription from some Pol II promoters.

*DLGAP3 PPI subnetwork* (3-set cluster) is named after the gene *DLGAP3* (Discs Large Homolog Associated Protein 3) that plays a role in the molecular organisation of synapses and neuronal cell signalling.

*Neurone recognition* (3-set cluster) is named after the GO biological process (GO:0008038) defined as “The process in which a neuronal cell in a multicellular organism interprets its surroundings”.

*WRB PPI subnetwork* (3-set cluster) is named after the gene *WRB* (Tryptophan Rich Basic Protein) also known as *CHD5* (Congenital Heart Disease 5) that has a potential role in the pathogenesis of Down syndrome congenital heart disease.

*Site of polarised growth* (3-set cluster) is named after the GO cellular component (GO:0030427) defined as “Any part of a cell where non-isotropic growth takes place”.

*Central nervous system neurone axonogenesis* (3-set cluster) is named after the GO biological process (GO:0021955) defined as “Generation of a long process from a neurone whose cell body resides in the central nervous system. The process carries efferent (outgoing) action potentials from the cell body towards target cells”.

*Regulation of neurone projection development* (6-set cluster) is named after the GO biological process (GO:0010975) defined as “Any process that modulates the rate, frequency or extent of neurone projection development. Neurone projection development is the process whose specific outcome is the progression of a neurone projection over time, from its formation to the mature structure. A neurone projection is any process extending from a neural cell, such as axons or dendrites (collectively called neurites)”.

*WDR37 PPI subnetwork* (7-set cluster) is named after the gene *WDR37* (WD Repeat Domain 37) that encodes a member of the WD repeat protein family and may facilitate the formation of heterotrimeric or multiprotein complexes.

*REACTOME_PI3K_EVENTS_IN_ERBB4_SIGNALING* (2-set cluster) is named after the Reactome pathway centred on genes involved in PI3K events in ERBB4 signalling. ERBB4 is a member of the Tyr protein kinase family and the epidermal growth factor receptor subfamily. It is for the normal development of the embryonic central nervous system, especially for normal neural crest cell migration and normal axon guidance.

*Regulation of skeletal muscle fibre development* (1-set cluster) is named after the GO biological process (GO:0048742) defined as “Any process that modulates the frequency, rate or extent of skeletal muscle fibre development”.

*HMGB2 PPI subnetwork* (1-set cluster) contains only one single gene set. It is named after the gene *HMGB2* (High Mobility Group Box 2) that encodes a member of the non-histone chromosomal high mobility group protein family.

**Significant tissue/cell types**

DEPICT determines the enrichment of expression in particular tissues and cell types by testing whether the genes overlapping the GWAS loci are highly expressed in any of 209 Medical Subject Heading (MeSH) annotations. Interestingly, all the significantly enriched tissues (FDR < 0.05) are related to the nervous system except retina, which is annotated to sense organs (**Supplementary Fig. 4b**). Furthermore, we observed only 1 significantly enriched cell-type, namely “*Neural Stem Cells*”. All the significantly enriched tissues and cell-type along with the gene names are listed in **Supplementary Data 7**.

**Significant gene prioritisation**

Any particular locus centred on a SNP may contain multiple genes. One straightforward approach is to nominate a gene that is closest to the SNP. But this approach does not consider if the expression of the gene is likely to be altered or regulated by the causal site in the locus. Therefore, we used DEPICT to map genes to associated loci, which prioritise important genes that share similar annotations in bioinformatic databases. For our 132 lead SNPs, DEPICT significantly prioritized (FDR < 0.05) 56 genes (**Supplementary Data 8**). For the two novel SNPs reported in this study (rs7336518 and rs7522116), DEPICT points to the *FOXO6* (Forkhead Box O6) and the *SLITRK1* (SLIT and NTRK Like Family Member 1) genes. *FOXO6* is predominantly expressed in the hippocampus and has been suggested to be involved in memory consolidation, emotion and synaptic function^13,14^. Similarly, *SLITRK1* is also highly expressed in the brain^15^, particularly in the frontal lobe, and has previously been suggested as a candidate gene for neuropsychiatric disorders^16^. In particular, *SLITRK1* is also associated with Tourette syndrome, which is characterised by persistent involuntary vocal and motor tics and often occurs together with Obsessive-Compulsive disorder and attention deficit hyperactivity disorder (ADHD)^17–19^.

# The GRAS data collection

All parts of GRAS data collection comply with the Helsinki Declaration and were approved by the ethical committee of the Georg-August-University of Göttingen (master committee) as well as by the respective local regulatory/ethical committees of all collaborating centres.

Subjects

GRAS schizophrenia and schizoaffective patients

The GRAS data collection consists of >1,200 deep phenotyped patients, diagnosed with SZ or schizoaffective disorder (SD) (according to *Diagnostic and Statistical Manual of Mental Disorders*, Fourth Edition Text Revision [DSM-IV-TR]) recruited across 23 collaborating centres across Germany^20,21^. All patients and/or their authorised legal representatives gave written informed consent. For the present study 1,067 patients were included, 67.1% were male (*n* = 716) and 32.9% female (*n* = 351). The average age was 39.46 ±12.58 years (range 17-79).

GRAS healthy controls

Healthy controls, who gave written informed consent, were voluntary blood donors, recruited by the Department of Transfusion Medicine of the George-August-University of Göttingen (Germany) according to national guidelines for blood donation. As such, they widely fulfil health criteria, ensured by a broad predonation screening process containing standardised questionnaires, interviews, haemoglobin, blood pressure, pulse, and body temperature determinations^20^. Participation as healthy controls for the GRAS data collection was anonymous, with information restricted to age, gender, blood donor health state and ethnicity. For the present study 1,169 subjects were included, 62.2% were male (*n* = 727) and 37.8% female (*n* = 442). The average age was 37.44 ±13.27 years (range 18-69).

Genotyping

Genotyping of the GRAS patients and control sample was done with a semi-custom Axiom myDesign genotyping array (Affymetrix, Santa Clara, CA, USA), based on a CEU (Caucasian residents of European ancestry from Utah, USA) marker backbone including 518,722 SNPs, and a custom marker set including 102,537 SNPs. The array was designed using the Axiom Design centre, applying diverse selection criteria^22^. Genotyping was done by Affymetrix on a GeneTitan platform. Several quality control steps were used (SNP call rate >97%, Fisher’s linear discriminant >3.6, heterozygous cluster strength off set > -0.1, and homozygote ratio off set > -0.9). These steps were completed with use of either genotyping console software (Affymetrix) or R. In a subsequent step, markers in X, Y, and mitochondrial chromosomes and those with Hardy–Weinberg equilibrium *P* < 1 × 10^-6^ (GRAS healthy controls) or *P* < 1 × 10^-10^ (GRAS patients) were removed, leaving 589,921 SNPs available for analyses.

Imputation and estimation of genetic principal components

The full details of genotype imputation and the estimation of the genetic principal components in the GRAS data collection are described elsewhere^23^. Briefly, imputation was done with the prephasing or imputation approach implemented in IMPUTE2 and SHAPEIT (chunk size 3 Mb)^24,25^. A version of the phase 1 integrated variant set release (v3) from the full 1000 Genomes Project dataset (March 2012) that is limited to variants with more than one minor allele copy (“macGT1”; Aug 26, 2012) was used as imputation reference dataset (INFO value > 0.1 and MAF >0.005). Ten principal components of the genetic data in the GRAS sample were obtained using the standard PGC protocol^23^.

Phenotyping procedures

Patients from the GRAS data collection were examined by the GRAS team of travelling investigators after giving written informed consent, own and/or authorised legal representatives. The GRAS team of travelling investigators consisted of 1 trained psychiatrist and neurologist, 3 psychologists and 4 medical doctors/last year medical students. All investigators had continuous training and calibration sessions to ensure the highest possible agreement on diagnoses and other judgments as well as a low interrater variability regarding the instruments applied. A full description of the GRAS data collection standard operating procedures is provided elsewhere^21^.

Deep clinical phenotyping data were available for all the GRAS patients. For the purpose of the present study the following phenotypes for SZ were selected: i) **age at prodrome** which precedes SZ onset and is characterized by cognitive decline, social withdrawal, and depression; ii) **age at disease onset** defined as onset of first psychotic episode; iii) **premorbid IQ** using the MWT-B (Mehrfachwahl-Wortschatz-Intelligenztest-B) which estimates the intellectual functioning of a person prior to known or suspected onset of disease^26^; iv) positive and negative symptoms using the **Positive and Negative Syndrome Scale (PANSS)**^27^. Each domain ranges from 7 to 49 with higher scores indicating severe symptoms; v) the **Global Assessment of Functioning (GAF)** which subjectively rates the social, occupational, and psychological functioning of an individual, e.g., how well one is meeting various problems-in-living, on a continuum ranging from 1 to 100^28^. Poorer functioning is indicated by lower GAF scores; vi) the **Clinical Global Impression of Severity (CGI-S)** scale which rates illness severity from 1 to 7 with higher scores indicating more severe illness^29^; vii) **years of education** was measured based on the highest degree obtained, converted into US-schooling year equivalents based on the 1997 International Standard Classification of Education (ISCED) of the United Nations^30^. Education was assessed retrospectively at the time of patient recruitment for the GRAS data collection. At this time, 75% of the GRAS patients were older than 29 years.

An important feature of SZ patients that may influence their everyday functioning and performance, and result in a considerable number of side effects, is their antipsychotic medication. Medication was assessed as the dose of present antipsychotic medication of each patient at the moment of the interview, expressed as chlorpromazine equivalents^31^.

The sociodemographic and clinical characteristics of the GRAS data collection are reported in **Supplementary Data 11**.

# Polygenic prediction of case-control status in the GRAS data collection

To investigate if our proxy-phenotype results help to predict SZ case-control status, we estimated a linear probability model (LPM) in Stata^32,33^. Genetic outliers (*n* = 13) based on self-reported non-European ancestry were excluded from all prediction analyses. Following the PGC protocol described in Ripke et al.^23^, we included 10 principal components as covariates (but no other variables). The proportion of variance explained (*Adjusted R*^2^) was computed by the comparison of a full model (covariates + PGS) to a reduced model (covariates only).

Results of the predictions with different models are summarised in **Supplementary Data 13a** and **14**. As effect sizes, we are reporting standardised regression coefficients. *SZ_132* scores significantly predict the case-control status in our replication sample ($P=5.4\times{10}^{-8})$ (**Supplementary Data 13a** Model 1) and remain significant even if we include *SZ_all* score as a control variable ($P=1.7\times{10}^{-4})$ (**Supplementary Data 13a** Model 3). The *EA_132*, *EA_all* and *Neuro_all* scores did not predict case-control status in our replication sample. The *BIP_all* score significantly predicts case-control status (**Supplementary Data 13a** Model 7), which is expected given the genetic correlation between BIP and SZ^1,34^. Interestingly, the *SZ_132* score still significantly predicts case-control status when all other scores are included as control variables (*P* $=3.4\times{10}^{-4})$ (**Supplementary Data 13a** Model 9), highlighting the importance of these 132 SNPs. Furthermore, as a robustness check we excluded the 132 SNPs from the construction of the *SZ_all* and *EA_all* scores. The prediction results did not change except a slight decrease of variance explained with *SZ_all* scores, which is expected given that 132 SNPs were excluded from the construction of the score (**Supplementary Data 13b**).

As an additional robustness check, we also ran a logistic regression with standardised PGS to predict SZ case-control status using the same explanatory variables as in Models 1-9 in **Supplementary Data 13a**. In all models, the *P* values of the coefficients were very similar to the ones obtained by LPM. In the equivalent of Model 9, we find that increasing the PGS from its mean by one standard deviation increases the probability of being an SZ case in the GRAS sample by 4.3% for the *SZ_132* score ($P=3.1\times{10}^{-4})$, 15.2% for the *SZ*_*all* score ($P=3.3\times{10}^{-26})$, and 7.1% for the *BIP_all* score ($P=4.1\times{10}^{-09})$, respectively.

**Supplementary Data 14** demonstrates that splitting the *SZ_all* score by sign concordance with EA (see **Supplementary Note 1**) does not significantly increase the predictive accuracy of SZ case-control status ($\Delta R^{2}=0.0002, P_{F}=0.213$).

# Phenotypic correlations in the GRAS data collection

Phenotypic correlations between years of education and different phenotypes of SZ in the GRAS data collection (see **Supplementary Note 5**) were calculated using Pearson's correlation. Target SZ phenotypes included were age at prodrome, age at disease onset, premorbid IQ, GAF, CGI-S, and positive and negative symptoms of the PANSS.

Results from the phenotypic correlations between the target phenotypes are reported in **Supplementary Data 15** and **Supplementary Fig. 7**. In summary, years of education was significantly correlated (all *P* values <0.0003) with all the quantitative traits of interest for the present study measuring the psychopathology and level of functioning of the patients (-0.212 < *r* < 0.435). The correlation was positive for age at prodrome and age at disease onset, indicating that the earlier the disease started the lower the level of education achieved. Years of education was also positively correlated with premorbid IQ and GAF and negatively correlated with CGI-S and PANSS positive and negative scores, indicating that higher levels of education were associated with less severe disease outcomes. Our results are in line with previous studies suggesting that less educated SZ patients are at higher risk of a poorer course of the disease^35^.

Moreover, we found a strong positive correlation between age at prodrome and age at disease onset (*r* = 0.918; *P* < 0.0001). Premorbid IQ was positively correlated with GAF (*r* = 0.200; *P* < 0.0001) and negatively correlated with CGI-S, PANSS positive and negative (-0.277 < *r* < -0.110; all *P* values < 0.0009). Measures of disease severity (CGI-S, PANSS positive, PANSS negative) were positively correlated with each other (0.478 < *r* < 0.638; all *P* values < 0.0001) and negatively correlated with an assessment of global functioning (GAF, -.579 < *r* < -0.824; all *P* values <0.0001), in line with previously reported results from other datasets^36^.

# Polygenic prediction of schizophrenia measures in the GRAS data collection

To investigate if our proxy phenotype results can predict specific SZ features, we predicted eight quantitative outcomes among the SZ cases in the GRAS data collection: years of education, age at prodrome, age at disease onset, premorbid IQ, GAF, CGI-S, PANSS positive and PANSS negative scores. These phenotypes are described in **Supplementary Note 5**.

SZ and BIP, although being classified as two different disorders, share several symptoms. The PANSS is a clinical instrument principally developed for use in SZ to identify the presence and severity of psychopathology symptoms. However, BIP patients also present some symptoms measured by this scale manifesting the phenotypic overlap between both diseases^37,38^. Thus, we used the sum score of the positive symptoms (delusions, conceptual disorganization, hallucinations, hyperactivity, grandiosity, suspiciousness/persecution, hostility) and the sum score of the negative symptoms (blunted affect, emotional withdrawal, poor rapport, passive/apathetic social withdrawal, difficulty in abstract thinking, lack of spontaneity and flow of conversation, stereotyped thinking) included in PANSS to test if the genetic associations we identified using our proxy-phenotype approach predict symptoms that SZ and BIP share.

**Baseline models without co-dependent polygenic risk scores**

For the prediction of each phenotype a linear regression model was used including the PGS described in the methods section of the main manuscript (*SZ_132, SZ_all, EA_132, EA_all, BIP_all and Neuro_all*). Each regression included 10 principal components as covariates. The regressions for the prediction of years of education and premorbid IQ also included the age of onset as a covariate. Furthermore, the regressions for the prediction of GAF, CGI-S and the PANSS scores also controlled for medication because medication significantly affects these outcomes. We calculated the marginal *R*^2^ of each PGS by squaring its standardised beta coefficient. Results of the predictions have been summarised in **Supplementary Data 16a**. We found that both the *EA_all* (*stand. beta* = 0.17, $P=8\times{10}^{-8}$) and the *EA_132* score (*stand. beta* = 0.08, $P=9\times{10}^{-3}$) were associated with years of education in the GRAS sample of SZ patients. However, the *EA_132* score did not survive correction for multiple testing (8 phenotypes and 6 PGS = 48 tests; thus the Bonferroni-adjusted *P* value is $\frac{0.05}{48}=1.04\times{10}^{-3}$. However, we note that neither the phenotypes nor the PGS are strictly independent. Therefore, the Bonferroni correction is likely to be too conservative to obtain a family-wide error rate of 0.05). Since age of disease onset, disease severity and progress of the disease can have an effect on the level of education achieved by a SZ patient, the years of education assessed in the GRAS data collection is not as solid as the measure used in the most recent EA GWAS.^2^ The potential measurement error of EA in the GRAS data collection may therefore lead to an underestimation of the predicted association. The *EA_all* score was also associated with premorbid IQ (*stand. beta* = 0.14, $P=1.8\times{10}^{-5}$). None of the PGS we constructed were associated with any of the other phenotypes tested (age at prodrome, age at disease onset, GAF, CGI-S, PANSS positive and negative scores) at $P<0.03$.

As a robustness check, we excluded the 132 EA lead SNPs from the proxy-phenotype analyses from the construction of the *EA_all* and *SZ_all* scores to correct for double-counting of these SNPs [*EA_all (without 132)* and *SZ_all (without 132)*]. All prediction models have been analysed again with these PGSs, yielding virtually identical results as our main model specification (**Supplementary Data 16b**).

## Co-dependent polygenic risk score prediction results

If heterogeneity in the genetic architecture of SZ subtypes is causing the observed enrichment of EA-associated loci with SZ, the sign concordance pattern of SNPs with both traits may contain relevant information that is pertinent to specific SZ symptoms (**Supplementary Note 1**). We tested this by constructing PGS that take the sign concordance of SNPs for both traits into account. Specifically, we took SNPs and SZ GWAS results that were used to construct the *SZ_all* score and sorted the SNPs into two sets based on their sign concordance with EA. The resulting two sets of SNPs were used to construct the *Concordant* and *Discordant* scores (see methods section of the main manuscript).

A linear regression model was used for the prediction of each phenotype. Following our statistical framework described in **Supplementary Note 1**, our baseline model predicted each phenotype using the full PGS for SZ and EA (*SZ_all*, *EA_all*). A second model regressed the phenotype on the *Concordant*, *Discordant*, and *EA_all* scores and we tested for improvement in model fit using the *F*-statistic. Each regression included the first 10 genetic principal components as covariates. The regressions for the prediction of years of education and premorbid IQ also included the age of onset as a covariate. Furthermore, the regressions for the prediction of GAF, CGI-S and the PANSS scores also controlled for medication because medication significantly affects these outcomes.

Results of the predictions are summarised in **Table 2**. As expected, the *EA_all* score was associated with years of education and premorbid IQ accounting for 3.12% ($P=4.4\times{10}^{-9}$) and 2.09% ($P=7.2\times{10}^{-6}$) of the variance in the baseline model, respectively. Interestingly, when we regressed on co-dependent PGS (*Concordant*, *Discordant*), we could also predict SZ symptoms such as GAF and PANSS negative (**Table 2**) to some extent. For example, splitting the *SZ_all* score into *Concordant* and *Discordant* yields an $\Delta R^{2}=0.63\%$ for PANSS negative and $\Delta R^{2}=0.39\%$ for GAF compared to the baseline model. Conditional on the effects of the *Concordant* and *Discordant* scores, the *EA_all* score is now associated with less severe disease outcomes, consistent with the observed phenotypic correlations. And conditional on the *EA_all* score, the *Concordant* score is now associated with better global function (GAF) and more severe negative symptoms (PANSS negative).

Clearly, the estimated coefficients of the *Concordant* and *Discordant* scores are not equal to each other and the *SZ_all* score, indicating genetic heterogeneity in the SZ symptoms (see **Supplementary Note 1**).

Using the *F-test* for comparing the two models (baseline and split models), the split model provides significant improvement compare to the baseline model for GAF ($p_{F}=0.023$) and PANSS negative ($p_{F}=0.007$) (**Table 2**), rejecting the null hypothesis that SZ is a genetically homogenous trait.

Results from linear regression models excluding the *EA_all* scores (**Supplementary Data 17**) suggest that the association between *EA_all* and disease outcomes is conditional on the effects of *Concordant* and *Discordant* scores. In the same way, the associations between *Concordant* and *Discordant* and disease outcomes are conditional on the effects of *EA_all* scores.

Although the correlation between *EA_all* and the *Concordant* (*r* = 0.278) and *Discordant* (*r* = -0.397) scores was only moderate (**Supplementary Data 12b**), we checked for possible multicollinearity in all our prediction models. The variance inflation factor was less than 3 for all the models, suggesting that multicollinearity is not a concern for the prediction results reported in **Table 2**^39^.

Since the GRAS data collection includes SZ and SD patients, we repeated the analyses described above excluding patients that were diagnosed with SD (*n* = 198). We found that the 95% confidence intervals of the estimated regression coefficients of both model specifications overlapped in all cases, implying that the genetic heterogeneity in SZ that we identify is not only due to SD (**Supplementary Data 18a** and **b**).

Moreover, we verified that randomly splitting the *SZ_all* score does not yield significant gains in predictive accuracy (**Supplementary Data 19**). We repeated the random split 100 times and checked how many times the increment in predictive accuracy is equal or higher than the observed $\Delta R^{2}$ (**Table 2**).

As an additional robustness check, we included years of education or premorbid IQ as covariates in the prediction analyses for GAF, CGI-S, PANSS positive and PANSS negative (**Supplementary Data 20a** and **b**). The increase in predictive accuracy from splitting the score by sign concordance (**Table 2**) is robust to controlling for these additional covariates, even though this leads to a reduction in sample size due to some missing observations (especially for premorbid IQ).

For highly polygenic traits such as SZ and EA, the predictive accuracy of PGS typically increases with the number of SNPs included in the scores^5,40^. Therefore, our main analyses are based on PGS derived from all SNPs that passed our quality control regardless of MAF (8,240,280 autosomal SNPs). However, as a robustness check we also constructed PGS including only SNPs with $MAF>1\%$ (i.e. dropping SNPs with allele frequency $\leq0.01$ or $\geq0.99$) in a first step and with $MAF>10\%$ (i.e. dropping SNPs with allele frequency $\leq0.10$ or $\geq0.90$) in a second step. We call the PGS resulting from these two steps *SZ_all_1%* (302,150 SNPs), *EA_all_1%* (306,977 SNPs), *Concordant_1%* (151,265 SNPs), *Discordant_1%* (150,885 SNPs) and *SZ_all_10%* (108,075 SNPs), *EA_all_10%* (106,608 SNPs), *Concordant_10%* (54,287 SNPs), *Discordant_10%* (53,788 SNPs), respectively. Results from these analyses are presented in **Supplementary Data 21a** and **b** indicating that our main conclusion that the polygenic prediction of clinical SZ symptoms can be improved by taking the sign concordance of loci for EA and SZ into account is robust to the exclusion of SNPs with low MAF for the calculation of the PGS.

We also conducted an alternative approach that maintains more signal in both *Concordant* and *Discordant* scores. Specifically, we first sorted all SNPs that pass quality control for both phenotypes (8,240,280 SNPs) based on sign concordance (4,147,926 SNPs with concordant and 4,092,354 SNPs with discordant signs) and then LD-prunes the resulting set, yielding 260,441 and 261,062 independent SNPs with concordant or discordant, respectively. We call the PGS resulting from this approach *Concordant_more_SNPs* and *Discordant_more_SNPs*. Using these scores yields much better prediction results for all measures of disease severity and SZ symptoms that we analyzed (**Supplementary Data 22**). In particular, the polygenic prediction of GAF measured in $\Delta R^{2}$ improves from 0.39% in the main model to 0.82% in the alternative specification. Similarly, predictive accuracy for PANSS positive and negative improves from 0.17% (*p*_F_  = 0.098) and 0.63% (*p*_F_ = 0.007) to 0.45% (*p*_F_ = 0.016) and 1.12% (*p*_F_ = 0.0004), respectively. Despite the better prediction results with the alternative approach, we still prefer our main approach (**Table 2**) because it is more conservative and a direct implementation of the statistical heterogeneity test we developed in **Supplementary Note 1**.

# Look-up of EA-associated lead SNPs in GWIS results schizophrenia – bipolar disorder

The “unique” SZ_(min BIP)_ results obtained from the GWIS were processed and merged with the EA GWAS results using the same procedures described in the methods section of the main manuscript (Proxy-phenotype method), leading to 1,153,214 SNPs that passed the quality control thresholds. This number is substantially lower than in the main look-up because the BIP GWAS was based on HapMap 2 imputation^41^ not on 1000 Genomes imputation^42^ like the SZ^23^ and EA GWAS^2^.

We repeated the clumping and look-up of the EA-associated lead SNPs in the cleaned and merged “unique” SZ_(min BIP)_ results following the steps described in in the methods section of the main manuscript (Proxy-phenotype method).

346 approximately independent EA lead-SNPs with *P*_EA_ < 10^-5^ were identified. None of them was significantly associated with “unique” SZ_(min BIP)_ after Bonferroni correction ($P=\frac{0.05}{346}=1.445\times{10}^{-4}$). **Supplementary Fig. 8** presents a Q-Q plot of this look-up. **Supplementary Data 23** reports the full results.

Sign concordance

We compared the signs of the beta coefficients of the 346 EA lead SNPs ($P_{EA}<{10}^{-5}$) with the beta coefficients of the “unique” SZ_(min BIP)_ results. If the signs were aligned, we assigned a “1” to the SNP and “0” otherwise. By chance, sign concordance is expected to be 50%. We tested if the observed sign concordance is different from 50% using the binomial probability test^43^. 154 of the 346 SNPs had the same sign (44.5%, *P* = 0.046, 2-sided). This result is consistent with a negative genetic correlation between the most strongly EA-associated SNPs and “unique” SZ_(min BIP)_ and contrasts with the positive genetic correlation between EA and SZ reported in Obkay et al.^2^.

Raw enrichment factor (not corrected for LD score of SNPs)

We calculated the raw enrichment factor of the EA-associated SNPs in the “unique” SZ_(min BIP)_ results using the approach described in the methods section of the main manuscript (Proxy-phenotype method). We obtained 109,188 approximately independent EA lead SNPs.

For $P_{EA}<{10}^{-5}$, we found 346 SNP ($\tau_{P_{EA}}=\frac{306}{109,188}=0.317\%$). For $P_{SZ}<0.05$, we found 6,190 SNP ($\tau_{P_{SZunique}}=\frac{6,190}{109,188}=5.669\%$). Thus, under the null hypothesis of no enrichment we expect $\left[ N_{S,EA\to SZunique} \right]=109,188 \times0.317\%\times5.669\%=20$ SNPs to have $P_{EA}<{10}^{-5}$ and $P_{SZunique}<0.05$ simulatenously. At these *P* value thresholds, we actually observe $N_{S,EA\to SZunique}=32$ SNPs, implying a raw enrichment factor of $\frac{32}{20}=1.6$. Thus, the observed enrichment of the EA-associated SNPs in the “unique” SZ_(min BIP)_ results is weaker than in our main look-up, but it still deviates from the expectation under the null hypothesis.

Raw enrichment *P* value (not corrected for LD score of SNPs)

We repeated the procedures described in the methods section of the main manuscript (Proxy-phenotype method) and found a raw enrichment *P* value of 0.02 ($Z = 2.317$, 2-sided). Thus, although the enrichment of the EA-associated top SNPs is unlikely to be drawn from the same distribution as all “unique” SZ_(min BIP)_ results with the same MAF distribution, the enrichment is weaker than in the main SZ GWAS results that did not control for the genetic overlap between SZ and BIP.

# Genetic correlations of GWAS and GWIS results

To test if the genetic overlap of SZ with EA is partially due to their genetic correlation with other traits, we computed genetic correlations of SZ and EA with three other phenotypes of particular relevance—BIP, childhood IQ, and neuroticism. Given that SZ is sometimes referred to as a cognitive disorder^44,45^, it is somewhat puzzling that previous studies did not find a significant (negative) genetic correlation between SZ and childhood IQ^1^. Furthermore, the personality trait neuroticism has been demonstrated to correlate across various psychiatric disorders and is positively associated ($r\approx0.4$) with a general psychopathology factor (*p*)^46^. In addition, moderate and strong negative genetic correlations of neuroticism have been reported for EA^2^ and depressive symptoms^7^, respectively, raising the possibility that neuroticism may contribute to the genetic overlap between EA and SZ. Finally, Nieuwboer et al.^47^ suggest that the genetic correlation between EA and SZ may be induced by the genetic correlation between SZ and BIP as well as the genetic correlation between BIP and EA. Extending this logic, it could also be that the lack of a clear negative genetic correlation between SZ and childhood IQ is induced by the genetic correlation between SZ and BIP.

Our analyses are facilitated by the fact that large-sample GWAS summary statistics are available in the public domain for all five traits (see data sources in **Supplementary Data 24**). In addition to analysing GWAS summary statistics, we also included the GWIS results described in the methods part of the main manuscript (GWIS SZ_(min BIP)_ and GWIS BIP_(min SZ)_).

**Supplementary Data 24** and **Fig. 3** display the results. When using the GWAS summary statistics, we obtain results very closely resembling those in earlier studies. In particular, we find a strong positive genetic correlation between SZ and BIP (*r*_g_ = 0.72, *P* = 8.57 × 10^-60^)^1,47^, a positive genetic correlations of EA with childhood IQ (*r*_g_ = 0.74, *P* = 2.22 × 10^-30^) and BIP (*r*_g_ = 0.27, *P* = 1.75 × 10^-11^), as well as a negative genetic correlation between EA and neuroticism (*r*_g_ = -0.25, *P* = 7.08 × 10^-17^)^2^. Also in line with previous reports is the insignificant genetic correlation between SZ and childhood IQ (*r*_g_ = -0.03, *P* = 0.61)^1^ as well as the positive genetic correlation between SZ and EA (*r*_g_ = 0.09, *P* = 1.04 × 10^-04^)^2^, both of which are counter-intuitive results. Furthermore, we find some positive genetic correlation of neuroticism with SZ (*r*_g_ = 0.19, *P* = 4.5 × 10^-07^) and BIP (*r*_g_ = 0.10, *P* = 0.06). However, these results are too weak to justify a GWIS approach that would purge the SZ and BIP results of their genetic correlation with neuroticism.

Interestingly, the genetic correlations change substantially when we purge the SZ results of their genetic overlap with BIP (GWIS SZ_(min BIP)_) and *vice versa* (GWIS BIP_(min SZ)_). The genetic correlations between EA and IQ with SZ_(min BIP)_ are now negative and significant (*r*_g_ = -0.16, *P* = 3.88×10^-04^ and *r*_g_ = -0.31, *P* = 6.00 × 10^-03^ respectively), which is more in line with the idea of SZ being a cognitive disorder^44^. Furthermore, the genetic correlations of EA and IQ with BIP(_min SZ_) remain positive and get somewhat stronger (*r*_g_ = 0.31, *P* = 2.87 × 10^-07^ and *r*_g_ = 0.33, *P* = 3.18 × 10^-02^ respectively). The genetic correlations of SZ_(min BIP)_ and BIP_(min SZ)_ with neuroticism, however, remain quite stable.

These results suggest two things: First, the genetic overlap between SZ and EA and the small, positive genetic correlation between the two traits is indeed to some extent caused by their genetic overlap with both BIP and childhood IQ (and not by their overlap with neuroticism). Second, once we purge the genetic association with SZ of their overlap with BIP, we see that the remaining part of “unique” SZ(_min BIP_) has *negative* genetic correlations with childhood IQ and EA. Thus, SZ diagnoses that have been used in large-scale GWAS analyses until now seem to comprise of at least two subtypes of the disease that have different genetic components: One part resembles a cognitive disorder which does not overlap with BIP, and one part does overlap with BIP but is not characterised by cognitive deficiencies.

# Simulating assortative mating

Previous studies found substantial assortative mating for EA, with spousal correlations in the range between 0.45 and 0.66, which led to a genetic resemblance among spouses for EA-associated genetic markers^48^. There is also evidence for substantial assortative mating for psychiatric disorders (in particular, SZ) with spousal correlations around 0.4^49^. One possibility is that strong, simultaneous assortative mating on EA and SZ may cause an enrichment of EA-associated loci for SZ. If this happens in the absence of phenotypic and genetic correlations between the two traits, one may call such an enrichment “spurious” because the genetic variants for EA would have no actual influence on SZ, even if they would be found to be robustly associated with SZ. We ran simulations to test how likely it is that our results may be driven by strong assortative mating.

Simulations

Description

We simulated an initial generation of *n* = 25,000. For each individual, two sets of 5,000 genetic markers were drawn from a binomial distribution with a 50% chance to be either 0 or 1. The sum of the two copies of each marker is the genotype of the individual. We assumed that two non-overlapping subsets of markers (500 each) were causal for either EA or SZ. We further assumed that both EA and SZ are 100% heritable, binary outcomes. The frequency of high EA was set to 0.3, and the frequency of SZ to 0.2. The phenotype of each individual was determined by examining the value of a PGS based on the known causal markers for each trait. We determined the relevant cut-off point of the score such that it matched the assumed frequencies of both traits in the population.

The initial generation went through a matching algorithm where each person was matched to a spouse and spousal correlations for both traits was assumed to be 0.6. Next, each couple had exactly two offspring and each offspring’s genotype was drawn from the genotypes of the parents. The offspring generation was then also matched to a spouse and the process was repeated for 50 generations. After each generation, we tested the causal EA markers for association with SZ and computed the raw enrichment test described in the methods section of the main manuscript (Proxy-phenotype method).

Our assumptions were not necessarily meant to represent the real world. Instead, our aim was to simulate a set-up with relatively extreme assumptions about heritability, assortative mating, and the prevalence of SZ, all of which would increase our chance of finding spurious enrichment. However, we note that our model was also deliberately simple: Implicitly, we assumed that there is no selective pressure on any genetic marker and that the effect sizes of the markers are constant over time. In reality, this may not be the case and both the phenotype and its genetic architecture may evolve.

Power

The power of our enrichment test is a function of the power to detect associations of individual genetic markers with SZ. To calculate power, we assumed an *OR* of 1.117. This *OR* was calculated using the fact that all causal markers had equal effect sizes and individual markers were drawn from a Bernoulli distribution, such that the PGS can be seen as a draw from a binomial distribution with parameters $n=1,000$ and $P=0.5$. As described above, this score was used to determine the SZ type. Specifically, a simulated individual had SZ if the score was larger than 513, which gives an overall chance of 20 percent to get SZ. The *OR* was calculated using the following formula:

$$P_{1}=P\left( SZ=1 \right| X_{SZ}=1), P_{0}=P\left( SZ=1 \right| X_{SZ}=0)$$

$$Odds=\frac{P_{1}(1-P_{0})}{P_{0}(1-P_{1})}$$

Where $P_{1}$ is the chance of getting SZ given that you have at least one of the causal variants increasing the chance of SZ, $X_{SZ}$, and $P_{0}$ is the chance of getting SZ given that you have the opposite variant. $P_{1}$ and $P_{0}$ can calculated from the binomial distribution. $P_{1}$ is the chance that a draw from a binomial distribution, with parameters $n=999$ and $P=0.5$, is larger than 512. $P_{0}$ is the chance that a draw from the same distribution is larger than 513.

We had 93.3% power to detect the causal markers associated with SZ at nominal significance and 18.1% after Bonferroni correction ($P=\frac{0.05}{5000}={10}^{-5}$). Furthermore, we had 80% power to detect (spurious) effects of $Odds\geq1.093$ at $P=0.05$ and of $Odds\geq1.182$ after Bonferroni correction. The raw enrichment test had even more power because it took the entire distribution of tested *P* values into account, not only the effects of one single SNP. Thus, we were well-powered to detect spurious enrichment in our simulation, even if the effects of individual genetic markers are relatively small.

Results

If assortative mating would cause the genetic overlap between EA and SZ, one would expect the absolute value of the *Z*-statistic from the enrichment test to increase over time. At some point, the difference between the two subsets would become statistically significant. The *Z*-statistic of the test for each generation is plotted in **Supplementary Fig. 9**. There is no persistent trend in the *Z*-statistics over time and the mean of the *Z*-statistics is not significantly different from 0 (*P* = 0.776). Furthermore, we cannot reject the hypothesis that the *Z*-statistics are simply drawn from a standard normal distribution (Shapiro-Wilk test, $P=0.075$, one-sided). Nevertheless, the *Z*-statistic drops below -1.96 in two simulated generations, indicating that the causal EA markers have lower *P* values than the non-causal markers. Yet, this result does not persist over time. In conclusion, it may be possible to find (spurious) enrichment in some generations by chance, but it is unlikely that assortative mating is a major cause for the strong genetic overlap that we observed between EA and SZ.

# Additional acknowledgments

**GRAS data collection**: The research of EU-AIMS receives support from the Innovative Medicines Initiative Joint Undertaking under grant agreement no. 115300, resources of which are composed of financial contribution from the European Union's Seventh Framework Programme (FP7/2007-2013), from the EFPIA companies and from Autism Speaks. We thank all subjects for participating in the study, and all the many colleagues who have contributed over the past decade to the GRAS data collection.

Niels Rietveld gratefully acknowledges funding from the Netherlands Organization for Scientific Research (NWO Veni grant 016.165.004).

Individual author contributions:

| First name | Last name | Conceptualization | Data Curation | Formal Analysis | Methodology | Project Administration | Resources | Software | Supervision | Visualization | Writing - Original Draft Preparation | Writing - Review & Editing |
| --- | --- | --- | --- | --- | --- | --- | --- | --- | --- | --- | --- | --- |
| Vikas | Bansal |  |  | X | X |  |  |  |  | X | X | X |
| Marina | Mitjans |  |  | X | X |  |  |  |  | X | X | X |
| Casper | Burik |  |  | X | X |  |  |  |  | X | X |  |
| Richard | Karlsson Linnér |  |  | X |  |  |  |  |  | X |  |  |
| Aysu | Okbay |  | X | X |  |  |  |  |  |  |  |  |
| Cornelius | Rietveld |  |  |  |  |  |  |  |  | X |  |  |
| Martin | Begemann |  | X |  |  |  |  |  |  |  |  |  |
| Stefan | Bonn |  |  |  |  |  | X |  | X |  |  |  |
| Stephan | Ripke |  | X | X |  |  |  |  |  |  |  |  |
| Ronald | De Vlaming |  |  | X | X |  |  |  |  | X | X |  |
| Michel | Nivard |  |  | X | X |  |  | X | X | X |  | X |
| Hannelore | Ehrenreich | X | X |  | X | X | X |  | X |  | X | X |
| Philipp | Koellinger | X | X | X | X | X | X |  | X |  | X | X |

References

1 Bulik-Sullivan BK, Finucane HK, Anttila V, Gusev A, Day FR, Consortium R *et al.* An atlas of genetic correlations across human diseases and traits. *Nat Genet* 2015; **47**: 1236–1241.

2 Okbay A, Beauchamp JP, Fontana MA, Lee JJ, Pers TH, Rietveld CA *et al.* Genome-wide association study identifies 74 loci associated with educational attainment. *Nature* 2016; **533**: 539–542.

3 Neale M, Cardon L. *Methodology for Genetic Studies of Twins and Tamilies*. Springer Science & Business Media, 1992.

4 Le Hellard S, Wang Y, Witoelar A, Zuber V, Bettella F, Hugdahl K *et al.* Identification of gene loci that overlap between schizophrenia and educational attainment. *Schizophr Bull* 2016. doi:10.1093/schbul/sbw085.

5 de Vlaming R, Okbay A, Rietveld CA, Johannesson M, Magnusson PKE, Uitterlinden AG *et al.* Meta-GWAS Accuracy and Power (MetaGAP) calculator shows that hiding heritability is partially due to imperfect genetic correlations across studies. *PLoS Genet* 2017; **13**: e1006495.

6 Rietveld CA, Esko TT, Davies G, Pers TH, Turley PA, Benyamin B *et al.* Common genetic variants associated with cognitive performance identified using the proxy-phenotype method. *Proc Natl Acad Sci U S A* 2014; **111**: 13790–13794.

7 Okbay A, Baselmans BML, De Neve J-E, Turley P, Nivard MG, Fontana MA *et al.* Genetic variants associated with subjective well-being, depressive symptoms, and neuroticism identified through genome-wide analyses. *Nat Genet* 2016; **48**: 624–633.

8 Frey BJ, Dueck D. Clustering by passing messages between data points. *Science (80- )* 2007; **315**.

9 Carbon S, Ireland A, Mungall CJ, Shu S, Marshall B, Lewis S *et al.* AmiGO: online access to ontology and annotation data. *Bioinformatics* 2009; **25**: 288–9.

10 Eppig JT, Blake JA, Bult CJ, Kadin JA, Richardson JE. The Mouse Genome Database (MGD): facilitating mouse as a model for human biology and disease. *Nucleic Acids Res* 2015; **43**: D726–D736.

11 Fabregat A, Sidiropoulos K, Garapati P, Gillespie M, Hausmann K, Haw R *et al.* The Reactome pathway Knowledgebase. *Nucleic Acids Res* 2016; **44**: D481–D487.

12 Stelzer G, Rosen N, Plaschkes I, Zimmerman S, Twik M, Fishilevich S *et al.* The GeneCards Suite: From gene data mining to disease genome sequence analyses. *Curr Protoc Bioinforma* 2016; : 1.30.1-1.30.33.

13 Salih DAM, Rashid AJ, Colas D, de la Torre-Ubieta L, Zhu RP, Morgan AA *et al.* FoxO6 regulates memory consolidation and synaptic function. *Genes Dev* 2012; **26**: 2780–2801.

14 Maiese K. FoxO Proteins in the Nervous System. *Anal Cell Pathol* 2015; **2015**: 1–15.

15 Aruga J, Yokota N, Mikoshiba K. Human SLITRK family genes: genomic organization and expression profiling in normal brain and brain tumor tissue. *Gene* 2003; **315**: 87–94.

16 Proenca CC, Gao KP, Shmelkov S V, Rafii S, Lee FS, Aruga J *et al.* Slitrks as emerging candidate genes involved in neuropsychiatric disorders. *Trends Neurosci* 2011; **34**: 143–53.

17 Carter AS, O’Donnell DA, Schultz RT, Scahill L, Leckman JF, Pauls DL. Social and emotional adjustment in children affected with Gilles de la Tourette’s syndrome: associations with ADHD and family functioning. Attention Deficit Hyperactivity Disorder. *J Child Psychol Psychiatry* 2000; **41**: 215–23.

18 Lombroso PJ, Scahill L. Tourette syndrome and obsessive–compulsive disorder. *Brain Dev* 2008; **30**: 231–237.

19 O’Rourke JA, Scharf JM, Yu D, Pauls DL. The genetics of Tourette syndrome: A review. *J Psychosom Res* 2009; **67**: 533–545.

20 Begemann M, Grube S, Papiol S, Malzahn D, Krampe H, Ribbe K *et al.* Modification of Cognitive Performance in Schizophrenia by Complexin 2 Gene Polymorphisms. *Arch Gen Psychiatry* 2010; **67**: 879.

21 Ribbe K, Friedrichs H, Begemann M, Grube S, Papiol S, Kästner A *et al.* The cross-sectional GRAS sample: A comprehensive phenotypical data collection of schizophrenic patients. *BMC Psychiatry* 2010; **10**: 91.

22 Hammer C, Zerche M, Schneider A, Begemann M, Nave K-A, Ehrenreich H. Apolipoprotein E4 carrier status plus circulating anti-NMDAR1 autoantibodies: Association with schizoaffective disorder. *Mol Psychiatry* 2014; **19**: 1054–6.

23 Ripke S, Neale BM, Corvin A, Walters JTR, Farh K-H, Holmans PA *et al.* Biological insights from 108 schizophrenia-associated genetic loci. *Nature* 2014; **511**: 421–427.

24 Delaneau O, Zagury J-F, Marchini JL. Improved whole-chromosome phasing for disease and population genetic studies. *Nat Methods* 2013; **10**: 5–6.

25 Howie BN, Donnelly P, Marchini J. A flexible and accurate genotype imputation method for the next generation of genome-wide association studies. *PLoS Genet* 2009; **5**: e1000529.

26 Lehrl S. *Mehrfachwahl-Wortschatz-Intelligenztest: MWT-B.* Spitta: Balingen, 1999.

27 Kay SR, Flszbein A, Opfer LA. The Positive and Negative Syndrome Scale (PANSS) for Schizophrenia. .

28 Association AP. *Diagnostic and statistical manual of mental disorders DSM-IV*. American Psychiatric Publishing, Inc., 1994.

29 Guy W. Clinical Global Impression (CGI). In: *ECDEU Assessment manual for psychopharmacology*. National Institute of Health, Psycho-pharmacology Research Branch.: Rockville, MD., 1976, pp 218–222.

30 Statistics UI for. International Standard Classification of Education. 2006.http://www.uis.unesco.org/Library/Documents/isced97-en.pdf (accessed 1 Jan2015).

31 Davis JM. Comparative Doses and Costs of Antipsychotic Medication. *Arch Gen Psychiatry* 1976; **33**: 858.

32 Wooldridge JM. *Econometric Analysis of Cross Section and Panel Data*. 2002 doi:10.1515/humr.2003.021.

33 Stata C. Stata Statistical Software: Release 14. 2015.

34 Purcell SM, Wray NR, Stone JL, Visscher PM, O’Donovan MC, Sullivan PF *et al.* Common polygenic variation contributes to risk of schizophrenia and bipolar disorder. *Nature* 2009; **460**: 748–752.

35 Levine SZ, Rabinowitz J. A population-based examination of the role of years of education, age of onset, and sex on the course of schizophrenia. *Psychiatry Res* 2009; **168**: 11–17.

36 Suzuki T, Uchida H, Sakurai H, Ishizuki T, Tsunoda K, Takeuchi H *et al.* Relationships between global assessment of functioning and other rating scales in clinical trials for schizophrenia. *Psychiatry Res* 2015; **227**: 265–269.

37 Bambole V, Johnston M, Shah N, Sonavane S, Desouza A, Shrivastava A. Symptom overlap between schizophrenia and bipolar mood disorder: Diagnostic issues. *Open J Psychiatry* 2013; **3**: 8–15.

38 Daneluzzo E, Arduini L, Rinaldi O, Di Domenico M, Petruzzi C, Kalyvoka A *et al.* PANSS factors and scores in schizophrenic and bipolar disorders during an index acute episode: a further analysis of the cognitive component. *Schizophr Res* 2002; **56**: 129–136.

39 Wooldridge JM. Multiple Regression Analysis: Estimation. In: *Introductory Econometrics: A Modern Approach*. Cengage Learning, 2013, pp 70–76.

40 Daetwyler HD, Villanueva B, Woolliams JA. Accuracy of predicting the genetic risk of disease using a genome-wide approach. *PLoS One* 2008; **3**: e3395.

41 Consortium TIH. A second generation human haplotype map of over 3.1 million SNPs. *Nature* 2007; **449**: 851–861.

42 The 1000 Genomes Project Consortium, Abecasis GR, Auton A, Brooks LD, DePristo MA, Durbin RM *et al.* An integrated map of genetic variation from 1,092 human genomes. *Nature* 2012; **491**: 56–65.

43 Sheskin D. The binomial sign test for a single sample. In: *Handbook of Parametric and Nonparametric Statistical Procedures*. Taylor & Francis Group: Boca Raton, 2007, pp 289–311.

44 Kahn RS, Keefe RSE. Schizophrenia Is a Cognitive Illness. *JAMA Psychiatry* 2013; **70**: 1107.

45 Kraepelin E. *Psychiatrie: Ein Lehrbuch für Studierende und Ärzte*. 4th ed. Verlag von Johann Ambrosius Barth: Leipzig, Germany, 1893.

46 Caspi A, Houts RM, Belsky DW, Goldman-Mellor SJ, Harrington H, Israel S *et al.* The p Factor. *Clin Psychol Sci* 2014; **2**: 119–137.

47 Nieuwboer HA, Pool R, Dolan CV, Boomsma DI, Nivard MG. GWIS: Genome-wide inferred statistics for functions of multiple phenotypes. *Am J Hum Genet* 2016; **99**: 917–927.

48 Hugh-Jones D, Verweij KJH, St. Pourcain B, Abdellaoui A. Assortative mating on educational attainment leads to genetic spousal resemblance for polygenic scores. *Intelligence* 2016; **59**: 103–108.

49 Nordsletten AE, Larsson H, Crowley JJ, Almqvist C, Lichtenstein P, Mataix-Cols D *et al.* Patterns of nonrandom mating within and across 11 major psychiatric disorders. *JAMA Psychiatry* 2016; **73**: 354.

1. This assumption will hold if the genetic variants, their effect sizes and their estimation errors have the same distribution in both subsets. [↑](#footnote-ref-1)
