# Supplementary material for: Genome-wide association study results for educational attainment aid in identifying genetic heterogeneity of schizophrenia
Source: Nat Commun. 2018 Aug 6;9:3078. doi: 10.1038/s41467-018-05510-z (PMC6079028; doi:10.1038/s41467-018-05510-z)
Supplement: Supplementary file 3 — Description of Additional Supplementary Files [file 41467_2018_5510_MOESM3_ESM.docx]

**Description of Additional Supplementary Files**

**File Name:** Supplementary Data 1

**Description:** Predictive accuracy of symptoms using trait-specific polygenic scores, in a simulation with endophenotypes affecting traits and symptoms heterogeneously.

**File Name:** Supplementary Data 2

**Description:** Schizophrenia association results for approximately independent SNPs that reached P value < 10^-5^ in the educational attainment GWAS.

**File Name:** Supplementary Data 3

**Description:** Overlap between the lead SNPs (or their LD partners, r^2^ > 0.5 and 250kb window) and SNP associations with other phenotypes listed in the NHGRI GWAS catalog.

**File Name:** Supplementary Data 4

**Description:** Pleiotropy analyses based on finemapping of GWAS results for EA and SZ using PAINTOR 3.0.

**File Name:** Supplementary Data 5

**Description:** DEPICT identified 111 significant reconstituted gene sets at FDR below 5%.

**File Name:** Supplementary Data 6

**Description:** Affinity Propagation method on the Pearson distance matrix for clustering of significant reconstituted gene sets.

**File Name:** Supplementary Data 7

**Description:** DEPICT identified 22 significant tissue/cell at FDR below 5%.

**File Name:** Supplementary Data 8

**Description:** DEPICT significantly prioritize 56 genes at FDR below 5%.

**File Name:** Supplementary Data 9

**Description:** HapMap 3 proxy-SNPs for the 132 SNPs that are jointly associated with EA (P < 10^-5^) and SZ (P < 0.05).

**File Name:** Supplementary Data 10

**Description:** LD-aware enrichment tests across different traits for SNPs jointly associated with EA (P < 10^-5^) and SZ (P < 0.05).

**File Name:** Supplementary Data 11

**Description:** Sociodemographic and clinical characteristics of the GRAS sample.

**File Name:** Supplementary Data 12

**Description:** Correlation between the different polygenic scores created in the GRAS sample a) including cases and controls b) including SZ cases only c) including healthy controls only.

**File Name:** Supplementary Data 13

**Description:** Polygenic risk score prediction of schizophrenia in the GRAS sample based on the 132 EA lead-SNPs from the proxy-phenotype analyses b) robustness check.

**File Name:** Supplementary Data 14

**Description:** Polygenic risk score prediction of schizophrenia in the GRAS sample based on concordant and discordant scores.

**File Name:** Supplementary Data 15

**Description:** Correlation between phenotypes among Schizophrenia cases in the GRAS sample.

**File Name:** Supplementary Data 16

**Description:** Polygenic risk score prediction of schizophrenia outcomes in the GRAS sample based on the 132 EA lead-SNPs from the proxy-phenotype analyses b) robustness check.

**File Name:** Supplementary Data 17

**Description:** Polygenic risk score prediction of schizophrenia outcomes in the GRAS sample (Table 2 in the manuscript excluding *EA_all* score).

**File Name:** Supplementary Data 18

**Description:** Polygenic risk score prediction of schizophrenia outcomes in the GRAS sample excluding schizoaffective patients a) (including *EA_all* score) b) (excluding *EA_all* score).

**File Name:** Supplementary Data 19

**Description:** Polygenic risk score prediction of schizophrenia outcomes in the GRAS sample by splitting SZ SNPs randomly into 2 halves and repeating 100 times.

**File Name:** Supplementary Data 20

**Description:** Polygenic risk score prediction of schizophrenia outcomes in the GRAS sample a) (robustness check - years of education as covariate) b) (robustness check - premorbid IQ as covariate).

**File Name:** Supplementary Data 21

**Description:** Polygenic risk score prediction of schizophrenia outcomes in the GRAS sample a) (PRS based on SNPs with MAF>1%) b) (PRS based on SNPs with MAF>10%).

**File Name:** Supplementary Data 22

**Description:** Polygenic risk score prediction of schizophrenia outcomes in the GRAS sample (alternative approach maintaining more SNPs in both *Concordant* and *Discordant* scores).

**File Name:** Supplementary Data 23

**Description:** Association results GWIS schizophrenia - bipolar disorder for approximately independent SNPs that reached P value < 10^-5^ in the educational attainment GWAS.

**File Name:** Supplementary Data 24

**Description:** Genetic correlations of GWAS and GWIS results that are central to the relationship between SZ and EA.
